# The immunoreceptor NKG2D promotes tumour growth in a model of hepatocellular carcinoma

**DOI:** 10.1038/ncomms13930

**Published:** 2017-01-27

**Authors:** Sam Sheppard, Joana Guedes, Anna Mroz, Anastasia-Maria Zavitsanou, Hiromi Kudo, Stephen M. Rothery, Panagiotis Angelopoulos, Robert Goldin, Nadia Guerra

**Affiliations:** 1 Department of Life Sciences, Imperial College London, SW7 2AZ London, UK; 2 Department of Cellular Pathology, Imperial College London, W2 1NY London, UK; 3 Facility for Imaging by Light Microscopy, Imperial College London, SW7 2AZ London, UK; 4 Department of Mathematics, National Technical University of Athens, Zografou, 15773 Athens, Greece

## Abstract

Inflammation is recognized as one of the drivers of cancer. Yet, the individual immune components that possess pro- and anti-tumorigenic functions in individual cancers remain largely unknown. NKG2D is a potent activating immunoreceptor that has emerged as an important player in inflammatory disorders besides its well-established function as tumour suppressor. Here, we provide genetic evidence of an unexpected tumour-promoting effect of NKG2D in a model of inflammation-driven liver cancer. Compared to NKG2D-deficient mice, NKG2D-sufficient mice display accelerated tumour growth associated with, an increased recruitment of memory CD8^+^T cells to the liver and exacerbated pro-inflammatory milieu. In addition, we show that NKG2D contributes to liver damage and consequent hepatocyte proliferation known to favour tumorigenesis. Thus, the NKG2D/NKG2D-ligand pathway provides an additional mechanism linking chronic inflammation to tumour development in hepatocellular carcinoma. Our findings expose the need to selectively target the types of cancer that could benefit from NKG2D-based immunotherapy.

## Abstract (web summary)

Expression of NKG2D immunoreceptor ligands on tumour cells is believed to inhibit tumour growth through engaging NKG2D-expressing immune cells. Here, the authors show that in a model of liver cancer the NKG2D/NKG2D-ligand pathway can also promote tumour formation by sustaining an inflammatory environment.

Immune checkpoint blockade therapy represents a major breakthrough in cancer treatment and attests the critical role played by immune cells in tumour surveillance^1^. Genetically engineered mice have been instrumental in validating the concept of tumour immunosurveillance as they provided direct evidence for the contribution of key immune components including *IFNG*, *RAG2*, *STAT1*, *TCRδ*, *PRF1* and *TNFSF10*, to antitumor responses mediated by both innate and adaptive effector cells^2^,^3^. The immunoreceptor NKG2D was added to the list of tumour suppressors a few years ago when we demonstrated that NKG2D-deficient mice were impaired in the surveillance of *de novo* malignancies^3^,^4^.

NKG2D is one of the most potent stimulatory receptors constitutively expressed on all NK cells; it is also present on subsets of invariant NKT and T cells^3^. NKG2D recognizes a large repertoire of ligands related to MHC class I molecules including MICA, MICB and the ULBP1-6 family of molecules in humans; MULT1 and several isoforms of RAE-1 and H60 in mice^5^. NKG2D ligands are self-proteins induced by stress pathways associated with infection, wounding and tumorigenesis^6^,^7^. As such, NKG2D ligands are generally absent from normal healthy tissues, albeit low levels of expression have been detected on certain tissues^8^. Several pathways regulate the expression of the various human and murine NKG2D ligands affecting their transcription, post-transcriptional stability and or ectodomain shedding^9^,^10^,^11^,^12^,^13^.

NKG2D-expressing cells are believed to reject neoplastic cells at early stages of tumorigenesis^3^, before the occurrence of immunoediting—the process by which tumour variants deprived of ligands evade immune surveillance^14^. While *in vitro* studies and *in vivo* transplanted tumour models have shown that NK cells and activated CD8^+^T cells can effectively reject tumour transfectants expressing NKG2D ligands^15^,^16^,^17^, evidence for NKG2D function in long-term models that recapitulate the complexity of the tumour microenvironment in human cancer are scarce^4^,^18^.

Interestingly, NKG2D has been shown to contribute to certain inflammatory disorders^19^,^20^, autoimmune diseases^21^,^22^,^23^,^24^ and wound associated inflammation^25^, which constitute a favourable ground for tumour initiation and progression. Indeed, chronic inflammation is now accepted as one of the hallmarks of cancer as it can provide pro-tumorigenic signals and subvert immunosurveillance^26^. A comprehensive understanding of the cell types, cytokines and chemokines involved in this process is far from elucidated and whether NKG2D aids the generation of pro-tumorigenic inflammation is not known.

The ability of NKG2D to promote inflammation and the sustained expression of NKG2D ligands observed during tumour progression in certain cancers^4^ led us to postulate that upon long-lasting injury, transforming cells would benefit from NKG2D-ligand expression in sustaining an inflammatory environment that promotes tumorigenesis.

Liver cancer is the third highest cause of cancer mortality worldwide, with a major prevalence in patients with underlying chronic liver disease and cirrhosis^27^. Several causative factors were identified including viral infection, metabolic disease, alcohol consumption and environmental chemicals^27^. Hepatocellular carcinoma (HCC) is by far the most common primary liver cancer, and typically develops from a background of chronic low-grade inflammation, characterized by a sequential progression from chronic liver injury to inflammation, hepatocellular necrosis and regeneration. Here we take advantage of the well-described model of chemically induced liver injury that closely mimics human HCC (ref. 28) to compare tumour development in NKG2D-sufficient and NKG2D-deficient mice. We demonstrate a novel outcome for NKG2D in its capacity to promote rather than delay tumour progression in the context of liver carcinogenesis.

## Results

### NKG2D enhances tumour progression in DEN-treated mice

**Figure 1:**
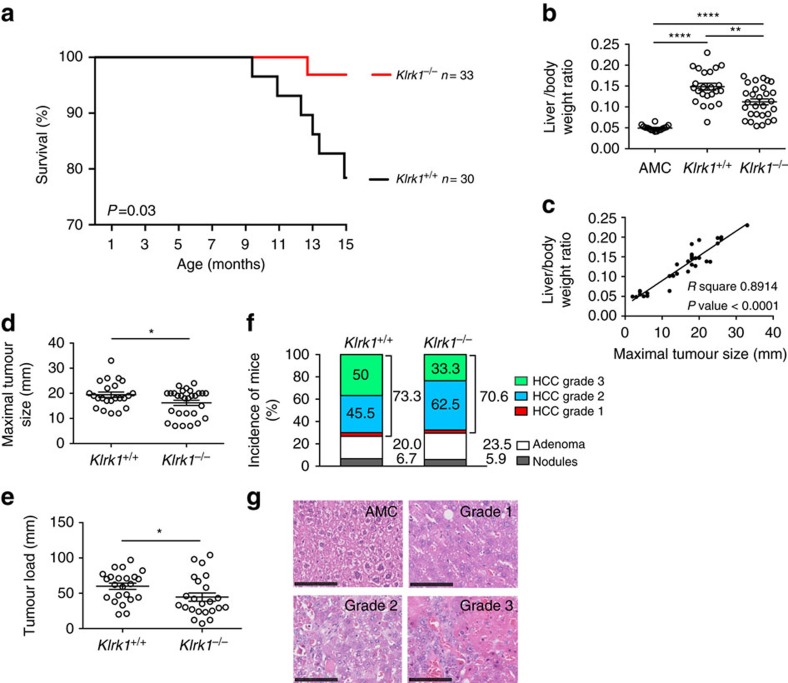
DEN-treated *Klrk1*^*+/+*^ mice display decreased survival and higher tumour burden in comparison to *Klrk1*^*−/−*^ mice. HCC was induced by a single intraperitoneal injection of diethylnitrosamine (DEN) (25 mg kg^−1^ body weight) at 14–21 days of age. (**a**) Kaplan-Meier representation of DEN-treated *Klrk1*^*+/+*^ (*n*=30) and *Klrk1*^*−/−*^ (*n*=33) mice survival with significant differences determined by log-rank test (*P*=0.03). (**b**) Liver/body weight ratio was assessed in 15-month-old *Klrk1*^*+/+*^, *Klrk1*^*−/−*^ mice treated with DEN and non-treated age-matched control mice (AMC). Statistical analysis was performed by unpaired Student's *t*-test. (**c**) Correlation between liver/body weight ratio and maximal tumour sizes in the liver of 15 months old *Klrk1*^*+/+*^ mice injected with DEN (*n*=30) (*P* value <0.0001 from linear regression analysis). (**d**–**e**) Maximal tumour size, measured macroscopically by diameter and Tumour load (sum of tumour diameter for all tumours with diameters >5 mm) in 15-month-old *Klrk1*^*+/+*^ and *Klrk1*^*−/−*^ mice treated with DEN. Graphs represent the mean±s.e.m. (*n*≥16). Statistical analysis was performed by unpaired Student's *t*-test. (**f**) Bar chart indicating the percentages of DEN-treated *Klrk1*^*+/+*^ (*n*=30) and *Klrk1*^*−/−*^ (*n*=34) mice showing nodule, adenoma and carcinoma (HCC). HCC are further divided according to histological grading. (**g**) Representative H&E staining of normal AMC liver and of liver tumours showing HCC of grade 1, 2 and 3 –defined as follow: Grade 1: Focal lesion with mildly increased nuclear-cytoplasmic ratio and/or a small increase in the number of mitoses, plus mild architectural distortion. Grade 2: Focal lesion with moderately increased nuclear-cytoplasmic ratio and/or a moderate architectural distortion. Grade 3: Focal lesion with marked increase in nuclear-cytoplasmic ratio and/or a marked increase in mitotic activity plus marked architectural distortion and/or necrosis. Scale bar represents 100 μm. Statistical analyses were performed using unpaired Student's *t*-test. Statistically significant differences between groups are denoted as: *P≤0.05, **P≤0.01, ****P≤0.0001.

The role of NKG2D in the development of cancer-linked to chronic inflammation was assessed in the widely used model of diethylnitrosamine (DEN)-induced hepatocellular carcinoma (HCC). A single injection of genotoxic DEN is administered to 14–21 days old male mice inducing the development of HCC after a latency period of 8–15 months^29^. Cohorts of DEN-treated *Klrk1*^*+/+*^ (NKG2D-WT) mice, *Klrk1*^*−/−*^ (NKG2D-KO) mice and untreated age-match control (AMC) mice were assessed over time for clinical signs of illness. We observed a significantly greater mortality following DEN administration in the presence of NKG2D, wherein 20% of *Klrk1*^*+/+*^ mice (6 of 30) versus 3% of *Klrk1*^*−/−*^ mice (1 of 33) (Log-rank test *P*=0.03) did not reach the experimental endpoint (Fig. 1a). In addition, of the mice that reached end point defined as 15 month of age, *Klrk1*^*+/+*^ mice displayed an increased tumour burden compared with *Klrk1*^*−/−*^ mice (Fig. 1b–e). Notably, *Klrk1*^*+/+*^ mice showed a significantly higher liver/body weight ratio (Fig. 1b), which positively correlated with the maximal tumour size (Fig. 1c). Accordingly, *Klrk1*^*+/+*^ mice displayed larger size tumours compared with *Klrk1*^*−/−*^ mice (Fig. 1d) and increased tumour load, measured as the sum of tumour diameters for all visible tumours over 5 mm (Fig. 1e).

The type and grade of tumour lesions were assessed to distinguish nodules and adenoma (non-HCC) from carcinoma (HCC) via histopathology and expression of HCC markers^30^. Carcinoma was the most represented lesion in both cohorts, with similar incidence in *Klrk1*^*+/+*^ *and Klrk1*^*−/−*^mice (73.3% versus 70.6% respectively) (Fig. 1f, Supplementary Table 1). Adenomas (20% of *Klrk1*^*+/+*^ versus 23.5% of *Klrk1*^*−/−*^) and nodules (6.7% of *Klrk1*^*+/+*^ versus 5.9% of *Klrk1*^*−/−*^) accounted for the tumours in the remaining mice, with similar incidence levels in both genotypes (Fig. 1f, Supplementary Table 1). Importantly, among HCC-bearing mice, a higher proportion of *Klrk1*^*+/+*^ than *Klrk1*^*−/−*^ mice (50% versus 33.3%) displayed a grade 3 HCC — the most advanced lesion based on histology (Fig. 1f,g; Supplementary Table 1) This phenotype confirms the advanced tumour burden (tumour size and liver/body weight ratio (Fig. 1b,d) observed in *Klrk1*^*+/+*^ mice. Immunohistochemical (IHC) scoring (Supplementary Table 2) was used to confirm the malignant nature of the tumours based on the expression of 3 markers: Glypican-3 (GPC3) — an oncofetal protein; Glutamine synthetase (GS) — an enzyme important for ammonia detoxification and glutamine synthesis and Heat shock protein 70 (Hsp70) — a peptide chaperone up regulated in stress conditions to prevent protein miss folding and aggregation^30^. Specifically, Glypican-3 and Hsp70 expression was increased and Glutamine synthetase became aberrantly expressed as tumours developed (Supplementary Fig. 1a,d,g). The overall IHC analysis supported the histological categorization of HCC versus non-HCC tumours, showing that HCC-bearing mice have higher IHC scores (Supplementary Fig. 1b,e,h; Supplementary Tables 1 and 2). Additionally, the incidence of *Klrk1*^*+/+*^mice developing tumours with the highest IHC score (score 3) was mildly increased compared with *Klrk1*^*−/−*^mice (19% versus 11.1% for GS; 9.6% versus 3.7% for Glypican-3 and 19% versus 11.1% for HSP70 respectively) (Supplementary Table 1; Supplementary Fig. 1c,f,i). Collectively, these results demonstrate that NKG2D has a substantial effect on tumorigenesis, accelerating the progression of hepatocellular carcinoma in DEN-treated mice, without altering the type of tumours that ultimately develop.

### NKG2D promotes CD8^+^T cell enrichment in the inflamed liver

**Figure 2:**
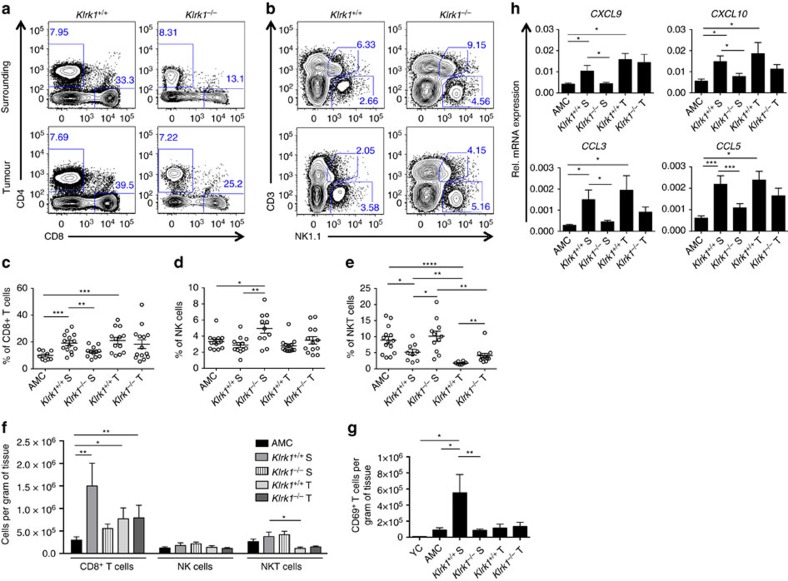
NKG2D favors the recruitment of CD8^+^ T cells to DEN-treated livers. (**a**) Representative flow cytometry plots of CD8 versus CD4 staining (**b**) NK1.1 versus CD3 staining and (**c**) percentages of CD8^+^T cells (CD8^+^CD4^−^NK1.1^−^CD3^+^), (**d**) NK (NK1.1^+^CD3^−^) and (**e**) NKT (NK1.1^low^CD3^low^) cells out of CD45^+^ cells, surrounding (S) and infiltrating tumours (T) in *Klrk1*^*+/+*^ and *Klrk1*^*−/−*^ DEN-treated mice and AMC. (mean±s.e.m.) where dots represent individual mouse. Statistical analysis was performed by unpaired Student's *t*-test. (**f**) Absolute numbers of CD8^+^T cells, NK and NKT cells per gram of surrounding tissue (S) and tumour tissue (T) in *Klrk1*^*+/+*^ and *Klrk1*^*−/−*^ DEN-treated, AMC and YC (*n*≥8 all groups). Graphs represent the mean±s.e.m. Statistical analysis was performed by Mann–Whitney test. (**g**) Absolute numbers of CD69^+^T cells per gram of surrounding tissue (S) and tumour tissue (T) in *Klrk1*^*+/+*^ and *Klrk1*^*−/−*^ DEN-treated, AMC and YC (*n*≥5 all groups). Graphs represent the mean±s.e.m. Statistical analysis was performed by unpaired Student's *t*-test. (**h**) Quantification of *CXCL9*, *CXCL10*, *CCL3* and *CCL5* mRNA transcript in livers of DEN-treated mice (*n*≥20) and AMC (*n*≥8). Graphs represent the mean±s.e.m. Statistical analysis was performed by unpaired Student's *t*-test. Statistically significant differences between groups are denoted as: *P≤0.05, **P≤0.01, ***P≤0.001, ****P≤0.0001.

Next, we characterized *ex vivo* the immune populations infiltrating tumours (T) and present in the non-neoplastic tissue surrounding tumours (S) of DEN-treated mice, compared with healthy liver from 15-month-old non-treated age-matched (AMC) and young control (YC) mice. We evidenced a drastic enrichment in CD8^+^T cells (Fig. 2a,c,f)—but not NK or NKT cells (Fig. 2b,d–f)—in HCC-bearing WT mice, with average CD8^+^T cell numbers of 1.5 × 10^6^ cells in the surrounding tissue and 7.7 × 10^5^ in the tumour, compared with 2.9 × 10^5^ cells per gram of tissue in livers from AMC mice (Fig. 2f). CD8^+^T cells accumulated in the surrounding tissue in an NKG2D-dependent manner, as demonstrated by the lower frequency and absolute numbers in *Klrk1*^*−/−*^ mice (13% and 5.5 × 10^5^ cells per g) compared with *Klrk1*^*+/+*^ littermates (19% and 1.5 × 10^6^ cells per g) (Fig. 2a,c,f). Thus, CD8^+^T cells represent the most abundant NKG2D-expressing cell type surrounding and infiltrating tumours.

A large majority of CD8^+^T cells present in all DEN-treated mice were identified as memory cells, due to the loss of the lymph-node homing receptor CD62L concomitant with high expression levels of lymphocyte function-associated antigen1 (LFA-1) and to the lack of expression of the proliferation marker Ki67 (Supplementary Fig. 2a,b). We further distinguished resident memory cells T_RM_—most of which are known to express the activating receptor CD69—from effector memory T_EM_ cells (mostly CD62L^-^CD69^-^). The number of CD69^+^T cells was significantly higher in NKG2D-WT than NKG2D-KO surrounding tissue (Fig. 2g) implying that the accumulation of T_RM_ likely accounts for the NKG2D-dependent enrichment of CD8^+^T cells within the surrounding tissue.

Importantly, chemokines known to help recruit immune cells to the damaged liver following viral infection and/or tissue injury^31^ were remarkably more represented in DEN-treated than AMC mice (Fig. 2h). Specifically, *CXCL9* (Mig), *CXCL10* (IP-10), *CCL3* (MIP1-α) and *CCL5* (RANTES) were significantly more expressed in the surrounding tissue of *Klrk1*^*+/+*^ than *Klrk1*^*−/−*^ mice (Fig. 2h), which could account for the NKG2D-dependent recruitment of memory CD8^+^T cells to the liver tissue. The corresponding receptors, namely *CXCR3* for *CXCL9* and *CXCL10* and *CCR5* for *CCL3* and *CCL5* were expressed in DEN-treated livers showing no significant differences between genotypes (Supplementary Fig. 2c). Altogether, these results illustrate that NKG2D contributes significantly to CD8^+^T cell enrichment in the inflamed liver tissue surrounding tumours, seemingly via increased chemotaxis of T_EM_ and persistence as T_RM_.

NKT cells represent a large fraction of lymphocytes residing in the liver. In DEN-treated mice, the number of NKT cell was decreased in the tumour tissue compared with the tumour surrounding area (Fig. 2f) regardless of NKG2D expression. This could be the result of an impaired ability of NKT cells to infiltrate the tumour bed. However, due to the gating strategy used to identify NKT cells (CD3^lo^ NK1.1^lo^), we cannot exclude that the decrease in NKT cell percentages within the tumours is partially caused by a possible downregulation of NK1.1 and CD3 upon NKT cell activation.

Regarding hepatic NK cells, similar proportions of immature and mature subsets, as identified by the expression of CD11b and CD27, were identified in all mice and tissues (Supplementary Fig. 3a). However, a drastic reduction in TRAIL-expressing NK cells (CD3^−^ NK1.1^+^) within the tumour was evidenced in both *Klrk1*^*+/+*^ and *Klrk1*^*−/−*^ mice (Supplementary Fig. 3b). At steady state TRAIL is expressed by liver-resident DX5^−^ NK cells. Co-staining of NK cells with TRAIL and DX5 showed that the TRAIL^+^ population was decreased due to a downregulation of TRAIL cell surface expression rather than an inability of DX5^−^ liver-resident NK cells to infiltrate the tumour (Supplementary Fig. 3c,d). These data suggest that tumour infiltrating NK cells may be impaired in their ability to induce TRAIL-mediated apoptosis against tumours, and/or to resolve inflammation by eliminating activated immune cells expressing the TRAIL receptor.

### CD8^+^T cells display a chronically activated phenotype

**Figure 3:**
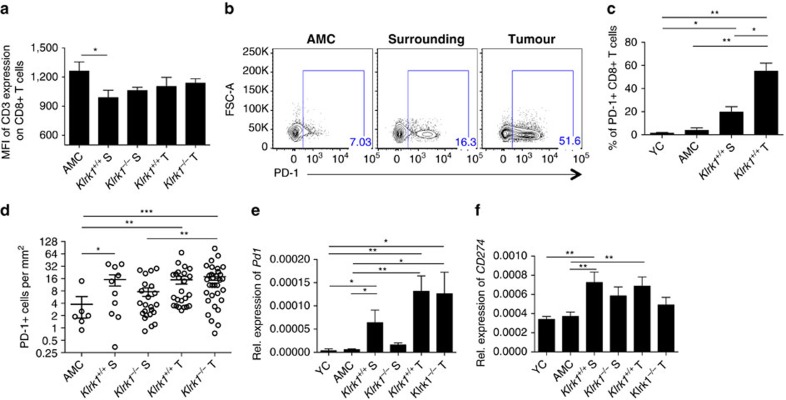
Hepatic CD8^+^T cells of DEN-treated mice have been chronically activated. (**a**) Median fluorescence intensity of CD3 expression on CD8^+^T cells (*n*≥10) within tumors (T) and surrounding (S) liver tissue of *Klrk1*^*+/+*^ and *Klrk1*^*−/−*^ DEN-treated mice and from AMC liver. (**b**) Representative flow cytometry plots of PD-1 expression *ex vivo* on CD8^+^T cells from DEN-treated *Klrk1*^*+/+*^ mice and AMC mice (representative of *n*≥3). (**c**) Percentages of CD8^+^T cells expressing PD-1 from DEN-treated *Klrk1*^*+/+*^ mice, age-matched control (AMC) mice and young controls (YC) (*n*≥3). Graphs represent the mean±s.e.m. (**d**) PD-1 positive cells per mm^2^ on liver tissue sections from 15-month-old *Klrk1*^*+/+*^ and *Klrk1*^*−/−*^ DEN-treated or AMC. Dots represent individual mice and show mean±s.e.m. Statistical analysis was performed by unpaired Student's *t*-test. (**e**,**f**) Relative expression of (**e**) *Pd1* and (**f**) *PD-L1* (*CD274*) mRNA transcripts, within tumours (T) and surrounding (S) liver tissue of *Klrk1*^*+/+*^ and *Klrk1*^*−/−*^ DEN-treated mice, AMC and YC (*n*≥9). Graphs represent the mean±s.e.m.

Next, we assessed the activation status of the CD8^+^T cell based on the CD3ɛ expression level as an indicator for CD3/TCR protein complex internalization upon MHC/epitope binding. CD3ɛ expression on CD8^+^T cell was reduced in all tissues from DEN-treated mice compared with AMC (Fig. 3a), the reduction being greatest on CD8^+^T cells localized in the surrounding tissue of tumour-bearing *Klrk1*^+/+^ mice (Fig. 3a).

PD-1, the inhibitory receptor and marker of exhaustion on activated CD8^+^T cells, is commonly induced upon chronic infection and cancer leading to CD8^+^T cell dysfunction upon engagement by its ligand PD-L1 (*CD274*) (ref. 32). As expected in this model of chronic inflammation and subsequent HCC, we observed a higher frequency of PD-1^+^CD8^+^T cells in the DEN-treated liver compared with AMC (Fig. 3b,c). Most likely due to exposure to tumour antigen within the tumour bed, the proportion of PD-1^+^CD8^+^T cells was greater amongst tumour infiltrating cells than surrounding cells, 55 versus 20% respectively (Fig. 3c). Absolute numbers of PD-1^+^ cells were as elevated in the surrounding tissue as in the tumour (Fig. 3d) reflecting the accumulation of CD8^+^T cells in the surrounding environment (Fig. 2f). Accordingly, numbers of PD-1^+^ cells in the surrounding tissue of *Klrk1*^*−/−*^ mice were lower than in *Klrk1*^*+/+*^, which was validated by the lower amount of transcripts for *Pd1* in the absence of NKG2D (Fig. 3e). Transcripts for PD-L1 were significantly more expressed in DEN-treated livers than controls (Fig. 3f) showing a non-significant trend towards a lower upregulation of transcripts in *Klrk1*^*−/−*^ mice. Other markers of exhaustion including *Lag-3*, *CTLA4*, *TIM3* and *BTLA* (ref. 32) were upregulated to different extents in DEN-treated mice compared with AMC (Supplementary Fig. 4). Altogether, these data imply that NKG2D contributes to the chronic activation of antigen-experienced CD8^+^T cells recruited to the DEN-damaged liver of NKG2D-WT mice.

### CD8^+^T cells exhibit partial downregulation of NKG2D

**Figure 4:**
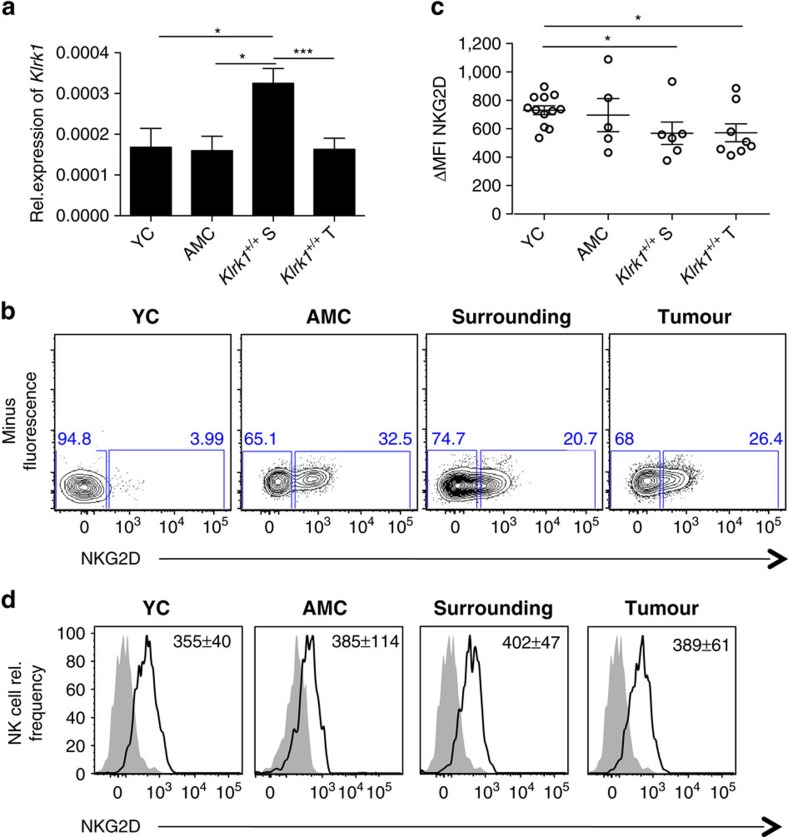
Partial downregulation of NKG2D on CD8^+^T cells but not NK cells. (**a**) Quantification of *Klrk1* mRNA transcripts in livers of DEN-treated mice (*n*≥22), YC and AMC (*n*≥5). (**b**) Representative flow cytometry plots of NKG2D expression *ex vivo* on CD8^+^T cells from DEN-treated mice (representative of *n*≥6), AMC and YC mice (representative of *n*≥5). Graphs represent the mean±s.e.m. (**c**) Delta median fluorescence intensity (MFI) of NKG2D expression on CD8^+^T cells from DEN-treated mice, AMC and YC. Dots represent individual mice. (**d**) Histograms of NKG2D expression on NK cells (black lines) from DEN-treated mice (representative of *n*≥6) and AMC (representative of *n*≥4). Fluorescence-minus-one control is shown as grey filled histogram. Values in each quadrant indicate the mean±s.e.m. of NKG2D Delta MFI staining.

NKG2D acts as a stimulatory receptor on NK cells and co-stimulatory receptor for the TCR/CD3 complex on activated T cells. Both cell types can downregulate cell surface NKG2D upon sustained engagement by cognate ligands^33^,^34^. In DEN-treated *Klrk1*^*+/+*^ mice, we observed a two-fold increase in total *Klrk1* transcript in surrounding tissues compared with control mice and tumour tissues (Fig. 4a). However, NKG2D expression on CD8^+^T cells present in the surrounding and tumour tissues was partially decreased compared with AMC and to the few NKG2D^+^T cells present in naïve liver (YC) (Fig. 4b,c). This was not the case for NK cells which maintained comparable levels of NKG2D in DEN-treated, AMC and YC livers (Fig. 4d). We conclude that the reduced expression of NKG2D on CD8^+^T cells is due to the chronic engagement of the receptor by its ligands.

### Sustained expression of NKG2D-ligands in DEN-treated livers

**Figure 5:**
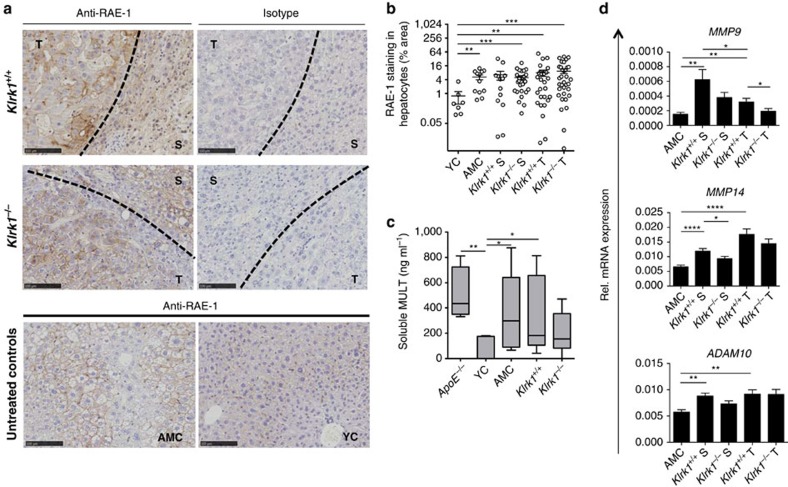
NKG2D ligands are expressed in DEN-treated livers. (**a**) Representative immunohistochemical staining of RAE-1 (brown) on hepatocellular carcinoma tissue sections showing cell-surface staining of hepatocytes within tumours (T) and surrounding tissue (S) of *Klrk1*^*+/+*^ (top left image) and *Klrk1*^*−/−*^ mice (middle left image) compared with isotype control staining (top and middle right image). RAE-1 staining of AMC (bottom left image) and 8-12 week old untreated young control mouse (YC) (bottom right image) are shown as indicated. A black dashed line demarcates boundary between tumour and surrounding tissue. DAB was used as revealing agent and eosin was used as a counter stain. Scale bar represents 100 μm. (**b**) Percentage area of RAE-1 positive hepatocytes on liver sections from 15-month-old *Klrk1*^*+/+*^ and *Klrk1*^*−/−*^ DEN-treated, age-matched control mice (AMC) or young control mice (YC). Graphs represent the mean±s.e.m. Statistical analysis was performed by unpaired Student's *t*-test. (**c**) ELISA of soluble MULT1 from the sera of *Klrk1*^*+/+*^ and *Klrk1*^*−/−*^ DEN-treated (*n*≥6), AMC, YC and *ApoE^-/-^* mice (*n*≥4). Absorbance values normalised to isotype controls. Boxes represent 25th–75th percentiles, whiskers represent minimum to maximum. Statistical analysis was performed by Mann-Whitney test. (**d**) Quantification of *MMP9*, *MMP14* and *ADAM10* mRNA transcript in livers of DEN-treated mice (*n*≥17) and AMC (*n*≥9). Graphs represent the mean±s.e.m. Statistical analysis was performed by unpaired Student's *t*-test. Statistically significant differences between groups are denoted as: *P≤0.005 **P≤0.01, ***P≤0.001, ****P≤0.0001.

The downregulation of NKG2D observed in CD8^+^T cells implies engagement by one or more NKG2D ligands. Immunohistochemistry on liver and tumour sections detected the presence of RAE-1 on the cell surface of transformed hepatocytes as well as on normal hepatocytes of the surrounding area (Fig. 5a,b). In line with our hypothesis proposing that NKG2D ligand expression is maintained rather than immunoedited in this type of tumour, we observed that RAE-1 was similarly expressed in both *Klrk1*^*+/+*^ and *Klrk1*^*−/−*^ mice (Fig. 5a,b). Also, consistent with a previous report^19^, RAE-1 was found expressed on normal untreated hepatocytes in AMC (Fig. 5a,b) indicative of a constitutive low-level inflammation likely to be derived from a constant exposure to gut-derived bacterial products. However, neither RAE-1 nor MULT1 was measurably expressed on CD11b^+^F4/80^+^ myeloid cells or leucocytes (Supplementary Fig. 5), unlike observations recently made in other tumour contexts^13^,^35^,^36^. Thus, our data show a persistent expression of cell surface RAE-1 on normal and transformed hepatocytes in the damaged liver.

Soluble forms of NKG2D ligands can be found in the serum of cancer patients including in HCC patients^6^. Notably, soluble MULT1 (sMULT1) is present at high levels in the serum of *ApoE*^*−/−*^ mice, which are prone to develop metabolic disorders associated with liver inflammation^13^. Here we observed elevated levels of sMULT1 in the serum of DEN-treated mice of both genotypes, at similar levels to those measured in *ApoE*^*−/−*^ mice (Fig. 5c). Fairly high levels of sMULT1 were detected in AMC sera indicating that part of sMULT1 expression is tumour independent, probably due to inflammation in age-related fatty livers.

Matrix metalloproteases (MMPs) are known to mediate proteolytic shedding of NKG2D ligands^9^ including MULT1 (ref. 13). Gene expression analysis showed that *MMP-9*, *MMP-14* and *ADAM-10* were upregulated in HCC-bearing mice (Fig. 5d), which likely contributes to MULT1 shedding from hepatocytes (Fig. 5c). These results imply that RAE-1 and MULT 1—in a membrane bound or soluble form —may therefore engage NKG2D in NKG2D-WT mice and further increase immune cell activation during tumour progression.

### CD8^+^T cells are functional and the main source of IFNγ

**Figure 6:**
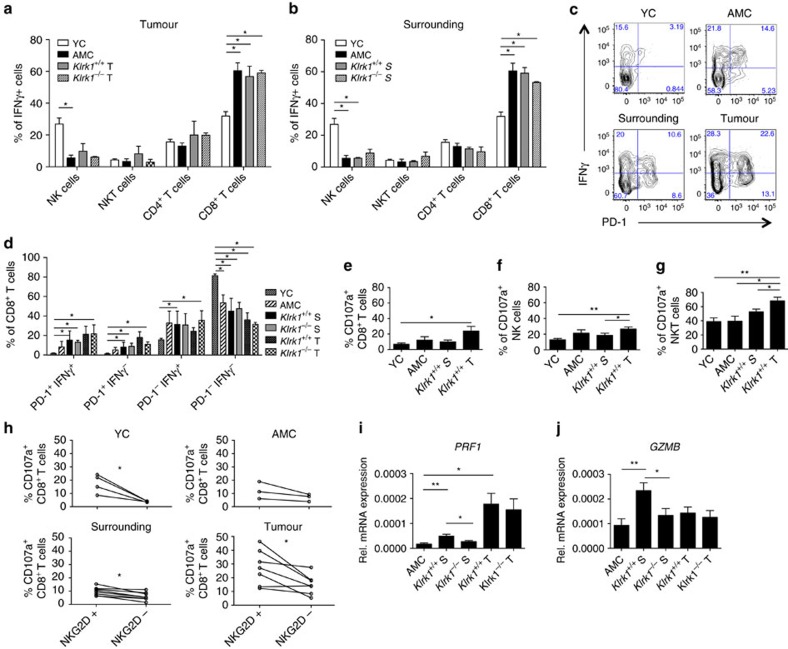
CD8^+^T cells are the main source of IFNγ in the tumour and surrounding milieu. (**a**) Percentages of NK, NKT, CD8^+^T and CD4^+^T cells among CD45^+^IFN-γ^+^ cells isolated from the tumour and (**b**) surrounding tissue of DEN-treated *Klrk1*^*+/+*^ and *Klrk1*^*−/−*^mice, AMC and YC (*n*=2-8) upon 4 h *in vitro* stimulation with PMA & Ionomycin. Graphs represent the mean±s.e.m. (**c**) Representative flow plots and (**d**) average percentages (mean±s.e.m.) of CD8^+^T cells expressing PD-1 and/or IFNγ post *in vitro* stimulation as described for (**a**). (**e**) Percentages of CD8^+^T, (**f**) NK and (**g**) NKT cells expressing CD107a (*n*=2–7). Graphs represent the mean±s.e.m. (**h**) Percentages of CD107a^+^ cells among CD8^+^T cells expressing NKG2D (NKG2D^+^) or not (NKG2D^−^) in DEN-treated *Klrk1*^*+/+*^ mice, AMC and YC. Dots represent populations from individual mice. Lines connect populations from the same mouse. (**i**) Relative expression of perforin and (**j**) granzyme B transcripts within tumour (T) and surrounding (S) tissue of *Klrk1*^*+/+*^ and *Klrk1*^*−/−*^ DEN-treated mice (*n*≥19) and AMC liver (*n*≥9). Graphs represent the mean±s.e.m. Statistical analyses were performed by unpaired Student's *t*-test. Statistically significant differences between groups are denoted as: *P≤0.05 and **P≤0.01.

The relative capability of each cell type to produce IFNγ was assessed in all NKG2D-expressing cell types. Among IFNγ-producing cells, CD8^+^T cells were the dominant source of IFNγ accounting for 56.8% of IFNγ^+^ lymphocytes within the tumour of *Klrk1*^*+/+*^ mice, while NK and NKT cells represented 9.8% and 8.1% respectively (Fig. 6a). This was further demonstrated when gating on individual cell types, where averages of 45, 22 and 4% of CD8^+^T cells, NK and NKT cells respectively produced IFNγ in tumours of NKG2D-WT mice (Supplementary Fig. 6a,b). Similar results were obtained from analysing the surrounding tissue (Fig. 6b; Supplementary Fig. 6a,b). The predominance of IFNγ^+^CD8^+^T cells over IFNγ^+^NK cells observed in AMC and DEN-treated mice, was not observed in naïve young control (YC) mice where proportions of IFNγ^+^CD8^+^T and IFNγ^+^NK cells were similar (Fig. 6a,b). This observation implies the occurrence of *in vivo* priming of hepatic CD8^+^T cells over time in 15-month aged mice.

Interestingly, IFNγ was produced by both PD-1^+^ and PD-1^−^ CD8^+^T cells in surrounding and tumour tissues (Fig. 6c,d). NKG2D-independent restimulation *in vitro,* using PMA/ionomycin, showed that CD8^+^T, NK and NKT cells from NKG2D-WT and NKG2D-KO mice had comparable percentages of IFNγ^+^ cells (Supplementary Fig. 6a,b) with comparable intensities of IFNγ fluorescence per cell (Supplementary Fig. 6c). Collectively, these results indicate that CD8^+^T cells in DEN-treated and AMC livers have been primed *in vivo* and remain capable of IFNγ production. CD8^+^T cells show similar intrinsic capacity to produce IFNγ in the presence or absence of NKG2D when stimulated under antigen-independent, NKG2D-independent conditions.

Next, we evaluated the cytotoxic potential of CD8^+^T, NK and NKT cells *ex vivo*, by means of perforin/granzyme B and CD107a expression as a measure of degranulation. Each cell type expressed CD107a to some extent in AMC livers and a significantly greater fraction of CD107a^+^ cells was seen among tumour infiltrating cells (Fig. 6e–g). Only 24% of CD8^+^T cells and 27% of NK cells expressed CD107a in NKG2D-WT tumours while 68% of NKT cells were CD107a^+^ in the same environment (Fig. 6e–g). The level of CD107a expression on cells positive for CD107a (median fluorescence intensity) was similar to that in control mice, indicating that the intrinsic capacity to degranulate was neither enhanced nor inhibited in the tumour and surrounding milieu (Supplementary Fig. 7a).

Similar to IFNγ production, a significant fraction (48% in the surrounding tissue and 62% in the tumour) of PD-1^+^ CD8^+^T cells expressed CD107a (Supplementary Fig. 7b) illustrating their overall competence to degranulate within the tumour. Interestingly, comparing NKG2D-expressing CD8^+^ T cells to NKG2D-negative CD8^+^ T cell subsets within the same NKG2D-WT mouse revealed that NKG2D-expressing cells are significantly more likely to have degranulated than NKG2D-negative CD8^+^T cells suggesting a higher cytotoxic potential in WT DEN-treated livers (Fig. 6h). This is supported by a significantly higher amount of perforin and granzyme B transcripts in *Klrk1*^*+/+*^ than *Klrk1*^*−/−*^ liver tissue surrounding tumours (Fig. 6i,j).

In conclusion, our data show that tumour development enhanced in NKG2D-WT mice strongly associates with a higher recruitment of effector CD8^+^T cells to the damaged liver. CD8^+^T cells displayed features of chronically activated cells as defined by the expression of PD-1 and downregulation of CD3 and NKG2D. A large majority of tumour infiltrating CD8^+^ PD-1^+^ T cells (63.3%, Fig 6c) are primed to produce IFNγ with the potential to deliver a lytic activity *in situ*.

### NKG2D exacerbates the local inflammation

**Figure 7:**
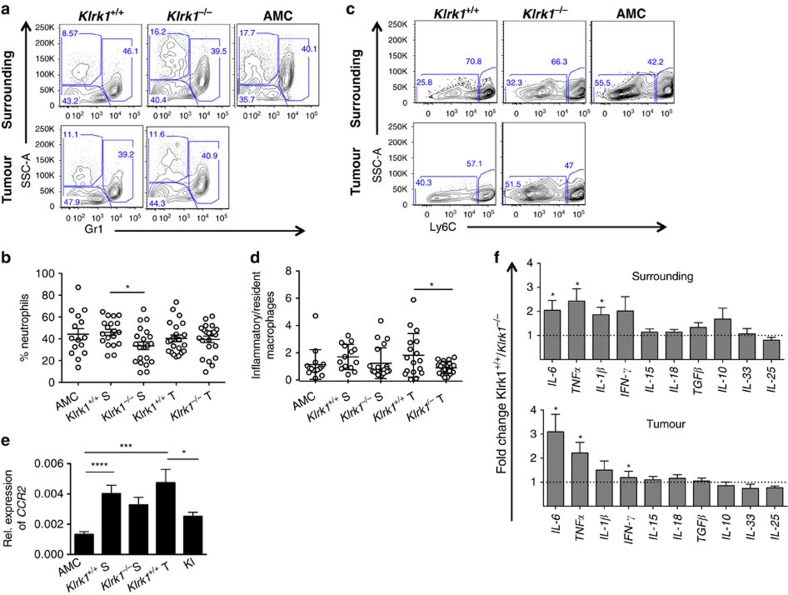
NKG2D exacerbates the local inflammation. (**a**) Representative flow cytometry plots of Gr1 staining versus SSC-A from CD11b^+^ gated liver cells of *Klrk1*^*+/+*^ and *Klrk1*^*−/−*^ DEN-treated mice (tumour and surrounding tissue) (representative of *n*≥19) and AMC (representative of *n*≥15). (**b**) Percentages of neutrophils (Ly6G^+^ or Gr1^hi^) out of CD11b^+^ cells surrounding (S) and infiltrating tumours (T) of *Klrk1*^*+/+*^ and *Klrk1*^*−/−*^ DEN-treated mice and from untreated AMC liver. Graphs represent the mean±s.e.m. (**c**) Representative flow cytometry plots of Ly6C versus SSC-A on CD11b^+^Ly6G^-^ F4/80^+^ cells from tumors and surrounding tissues of *Klrk1*^*+/+*^ and *Klrk1*^*−/−*^ DEN-treated mice and untreated AMC liver are shown (representative of *n*≥15). (**d**) Ratio of inflammatory (Ly6C^hi^ or Gr1^intermediate^) versus resident (Ly6C^lo^ or Gr1^lo^) macrophages (CD11b^+^, F4/80^+^, Ly6G^−^ or Gr1^lo.int^) from tumours (T) and surrounding (S) tissue of *Klrk1*^*+/+*^ and *Klrk1*^*−/−*^ DEN-treated mice and untreated AMC. Graphs represent the mean±s.e.m. (**e**) Relative expression of *CCR2* mRNA transcripts detected in tumours (T) and surrounding (S) tissue of *Klrk1*^*+/+*^ and *Klrk1*^*−/−*^ DEN-treated mice (*n*≥19) and in AMC livers (*n*≥11). (**f**) Fold change in expression of *IL-6, TNFα, IL-1β, IFN-γ, IL-15, IL-18, TGFβ, IL-10, IL-33* and *IL-25* transcripts in tumours (bottom) and surrounding liver tissue (top) of DEN-treated *Klrk1*^*+/+*^ over *Klrk*^*−/−*^ (*n*=9–22). Graphs represent the mean±s.e.m. All statistical analyses were performed by unpaired Student's *t*-test. Statistically significant differences between groups are denoted as: *P≤0.005, ***P≤0.001, ****P≤0.0001.

In addition to lymphocytes, circulating myeloid cells are rapidly recruited to inflamed tissue where they can aid tissue repair in addition to mediating either inflammatory or regulatory responses. Therefore, we examined the CD45^+^ CD11b^+^ population for an indirect effect of NKG2D on the myeloid compartment and observed imbalanced frequencies of the main cell types present in the tumour microenvironment. Notably, *Klrk1*^*−/−*^ mice exhibited a significant decrease in CD11b^+^ GR1^hi^ neutrophils (Fig. 7a,b) and slight increase in CD11b^+^/GR1^lo^/SSC^hi^ eosinophils compared with *Klrk1*^*+/+*^ littermates in the surrounding tissue (Fig. 7a). Using Ly6C as a marker to distinguish resident versus inflammatory macrophages, we observed an increased percentage of inflammatory Ly6C^hi^ macrophages and reduced percentage of resident Ly6C^lo^ macrophages in *Klrk1*^*+/+*^ versus *Klrk1*^*−/−*^ mice resulting in a predominance of inflammatory macrophages within the tumour (Fig. 7c,d). *CCR2*, a chemokine receptor preferentially involved in monocytes trafficking to inflamed tissues and that defines their differentiation into pro-inflammatory macrophages^31^, was significantly more expressed in the tumours from *Klrk1*^*+/+*^ than *Klrk1*^*−/−*^ mice (Fig. 7e) implying a better recruitment of circulating monocytes and/or macrophages in the presence of NKG2D.

Next, we performed a gene expression analysis of key pro-inflammatory and anti-inflammatory cytokines, known to constitute the tumour microenvironment. These included IL-6 and TNF-α whose deregulation is directly connected with the pathogenesis of several chronic inflammatory disorders in addition to cancer^37^,^38^. IL-6 in particular is known to be a main driver of carcinogenesis in HCC (ref. 38). The expression levels of several cytokines including IL-6, TNF-α, IL-1β and IFN-γ were at least 2 fold higher in the surrounding tissue of *Klrk1*^*+/+*^ than *Klrk1*^*−/−*^ mice (Fig. 7f upper panel). IL-6 and TNF-α were also more expressed within the *Klrk1*^*+/+*^ tumour (Fig. 7f lower panel) demonstrating that NKG2D has a significant influence on the tumour microenvironment, boosting the production of key pro-inflammatory cytokines. This conclusion is supported by the results from MMP expression analysis (Fig. 5d). In addition to their function in matrix remodelling, MMPs are potent regulators of acute and chronic inflammation as they play an important role in the recruitment of immune cells to the tumour microenvironment. Several MMPs including MMP-9 exert proteolytic cleavage of pro-TNF-α−the membrane bound precursor of soluble TNF-α− expressed on macrophages and T cells and involved in skin carcinogenesis^39^. Greater amounts of MMP mRNA transcripts were detected in *Klrk1*^*+/+*^ compared with *Klrk1*^*−/−*^ mice (Fig. 5d) and thus potentially contributed to amplify inflammatory responses and/or lymphocyte recruitment in *Klrk1*^*+/+*^ mice.

### NKG2D promotes hepatic injury and compensatory proliferation

**Figure 8:**
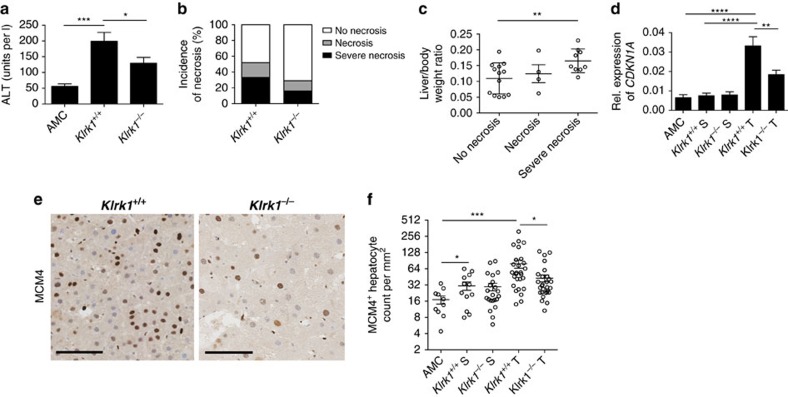
Hepatocyte cell death and proliferation induced by DEN treatment are increased in the presence of NKG2D. (**a**) Serum alanine aminotransferase (ALT) levels in 15-month-old *Klrk1*^*+/+*^ and *Klrk1*^*−/−*^ DEN-treated (*n*≥17) and untreated AMC (*n*≥5) mice. Graphs represent the mean±s.e.m. (**b**) Incidence of necrosis was detected by TUNEL staining on tissue sections from *Klrk1*^*+/+*^ and *Klrk1*^*−/−*^ DEN-treated mice (*n*≥12). (**c**) Liver/body weight ratio of *Klrk1*^*+/+*^mice showing no necrosis, necrosis or severe necrosis by TUNEL assay. Graphs represent the mean±s.e.m. (**d**) Relative expression of *CDKN1A* (p21) mRNA transcripts in livers of *Klrk1*^*+/+*^ and *Klrk1*^*−/−*^ DEN-treated (*n*≥19) and AMC (*n*≥10). Graphs represent the mean±s.e.m. (**e**) Representative staining of proliferating hepatocytes by MCM4 (brown) with hematoxylin counterstained (blue). Black scale bar represents 100 μm. (**f**) MCM4 positive cells per mm^2^ of liver tissue (*n*≥10). Graph represents the mean±s.e.m. Statistical analysis was performed by unpaired Student's *t*-test, where statistically significant differences between groups are denoted as: *P≤0.05, **P≤0.01, and ***P≤0.001, ****P≤0.0001.

The link between liver injury, death-driven proliferation and tumour progression is very well-described in HCC^40^. Compared with non-treated AMC, DEN-treated mice of both genotype showed elevated level of serum alanine aminotransferase (ALT), a marker of liver injury known to be elevated in high grade HCC. *Klrk1*^*+/+*^ mice displayed significantly higher concentrations of ALT than *Klrk1*^*−/−*^ littermates, signifying increased liver damage in the presence of NKG2D (Fig. 8a). Concomitantly, the prevalence of necrotic hepatocytes in the tumour was evaluated via TUNEL assay. Necrotic areas were greater in *Klrk1*^*+/+*^ compared with *Klrk1*^*−/−*^ mice and significantly correlated with a higher tumour burden (Fig. 8b,c). These results illustrate a role for NKG2D in sustaining liver damage and death of hepatocytes, known to trigger compensatory proliferation and tissue regeneration^26^,^40^. Indeed, compared with *Klrk1*^*−/−*^, tumours in *Klrk1*^*+/+*^ mice proved to express a substantially higher level of the cyclin-dependent kinase inhibitor *CDKN1A* (p21) (Fig. 8d), a critical player in liver regeneration and hepatocarcinogenesis^41^. Thus, hepatocyte proliferation was evaluated by expression of MCM4, a marker of DNA synthesis. Representative histological sections shown in (Fig. 8e) exhibited a strong nuclear staining for MCM4, showing a significantly higher number of proliferating hepatocytes in the presence of NKG2D (Fig. 8e,f). Our data illustrate the impact of NKG2D in promoting liver damage and subsequent hepatocytes proliferation associated with a greater tumour burden.

## Discussion

The importance of the NKG2D/NKG2D-ligand pathway in regulating immune responses is now well-established. Yet, the extent to which NKG2D protects against or promotes tissue injury and neoplastic transformation in physiological settings remains poorly understood. Here, we provide the first demonstration that the NKG2D/NKG2D-ligand pathway promotes liver carcinogenesis. Our results directly implicate NKG2D in the establishment of a tissue environment that favors CD8^+^T cell recruitment, exacerbates the local pro-inflammatory responses and increases liver damage resulting in a higher tumour burden. These conclusions are directly supported by studies from Huang *et al*. demonstrating that inactivation of all known murine NKG2D by multiple ligands on hepatocytes, using a triple RAE-1/H60/MULT1 shRNA-expressing vector, reduces dramatically IFN-γ and TNF-α mediated liver injury induced in mice^42^.

Our finding seems contradictory to the established paradigm that NKG2D acts as a tumour suppressor. Yet, coexistence of anti-tumour and pro-tumour responses is plausible and supported by studies using models of MCA-induced fibrosarcoma^43^, skin papilloma^44^,^45^ and inflammation–associated lung tumours^46^. We previously provided evidence for an NKG2D-mediated surveillance of primary tumours using two independent mouse models: the EμMyc transgenic mouse developing B-cell lymphoma and the transgenic TRAMP mouse as a model of prostate cancer^3^,^4^. In NKG2D-sufficient TRAMP mice, early aggressive tumours were efficiently rejected except for the few that lost NKG2D-ligand expression. Interestingly, a second type of prostate tumour, less aggressive and arising later in TRAMP mice, was not rejected by NKG2D-expressing cells despite maintaining high levels of cell surface NKG2D ligands. Though not statistically significant, these less aggressive tumours tended to arise earlier in NKG2D-sufficient than NKG2D-deficient mice, suggesting that their progression was accelerated to some extent in the presence of NKG2D (ref. 4). Similarly, slow-growing fibrosarcomas, induced by a low-dose of the MCA carcinogen, showed high levels of cell surface NKG2D-ligand and a trend to develop earlier in NKG2D-sufficient than NKG2D-deficient mice^4^. Whether NKG2D also display a protective anti-tumour function at onset and/or during HCC progression in the present model remains to be established. Yet, common features of the HCC model, the late-arising prostate tumours and the low-dose MCA-induced tumours model are: (i) the slow pace of tumour progression (ii) the high level of immune cells infiltrating the tumours and (iii) the sustained expression of NKG2D ligands. These observations imply that if the NKG2D/NKG2D-ligand pathway fails to efficiently reject early neoplastic lesions, it may ultimately be coerced to favour tumour growth.

During tumour development, the function of NKG2D-expressing cells is likely to evolve over time, depending on (i) the composition of the inflammatory microenvironment, (ii) the level of expression of cell surface/soluble ligands and (iii) the expression level of NKG2D which can be reduced upon sustained triggering, consequently leading to the desensitization of NK cells^13^,^18^,^47^. Evidence of T cells desensitization exists^33^,^34^ but it seems to depend on the antigenic stimulation^18^,^48^ and/or the inflammatory context^22^,^49^. In the present HCC model, NKG2D was partially downregulated on CD8^+^T cells but not on NK cells. A significant amount of both CD8^+^T and NK cells have remained competent as shown by *ex vivo* expression of CD107a and their ability to produce IFNγ, arguing against immune desensitization in this environment. This could be explained by the lack of desensitizing RAE-1 expression on myeloid cells or the presence of sMULT1 in the serum of DEN-treated mice, preventing hypothetical desensitization via RAE-1-expressing tumours. Additionally, the inflammatory milieu could act to maintain the activity of some effector CD8^+^T cells in an NKG2D-dependent yet antigen-independent manner^22^,^49^ or in an NKG2D-independent manner as shown in the context of viral infection^48^.

The long-term nature of this HCC model preclude us from directly demonstrating by means of antibody depletion and/or adoptive transfer which cell type is responsible for the observed pro-tumour effect. A role for CD8^+^T cells is supported by findings in studies of skin^50^, as well as liver carcinogenesis^51^. Indeed, Wolf and colleagues conclusions raised in a model of liver carcinogenesis are highly relevant to our studies in showing that infiltration and activation of CD8^+^T cells resulted in liver damage linked to the development of nonalcoholic steatohepatitis-induced HCC (ref. 51). Importantly, RAE-1 overexpression has been shown to promote the infiltration of unconventional αβT cells to the skin tissue^45^ and of conventional CD8^+^T cells in the contexts of transplanted tumour^17^ and autoimmune insulitis^52^. Specifically, the development of insulitis observed by Marckiewicz and coll. in a transgenic mouse model of Rae-1-expressing β-islet cells directly support our findings in showing that NKG2D helps recruit antigen-specific T cells to the pancreas^52^. They also show increased secretion of CCL5, a chemokine that is believed to favour further CTL recruitment to the pancreas^52^. Expression of CCL5 was upregulated in our studies along with other T cell chemoattractants namely CXCL9, CXCL10, CCL3 —all of which have shown increased expression in liver carcinoma^53^. Further studies are needed to elucidate the mechanisms by which NKG2D indirectly promotes tumorigenesis in this model besides helping recruit CD8^+^T cells to damaged liver and consequently further the amount of IFNγ in the milieu.

Here, we propose a model supporting a dual function for NKG2D in tumour immunity. In precancerous lesions, NKG2D-mediated tumour elimination is likely to be dominated by innate immune cells such as NK and NKT cells due to their independence from the need for Ag recognition^54^. In eliminating damaged cells they likely contribute to shaping an innate inflammatory environment—rich in myeloid cells and chemokines including Mip1-α, CXCL9, CXCL10 and CCL5- that drive the recruitment and activation of CD8^+^T cells. Thus, under continuous exposure to NKG2D ligands, activation of CD8^+^T cell and potentially NK and NKT cells could continuously feed the pro-inflammatory milieu via directly producing inflammatory components including TNFα, IFNγ and MIP1-α or indirectly via helping myeloid cells to further the inflammatory milieu with IL-6, TNFα and IL-1β. Direct killing of neoplastic cells by some CD8^+^T, NKT and/or NK cells capable of degranulation can further contribute to liver damage, and with it the release of pro-inflammatory factors by dying hepatocytes. In this context, cycles of persistent hepatic injury, hepatocyte death and consequential liver regeneration, which encompasses the proliferation of mutated hepatocytes, ultimately promotes tumour growth. Nonetheless, we cannot exclude that a fraction of ‘exhausted' CD8^+^T cells functionally impaired in their anti-tumour function, can indirectly favour tumour growth in NKG2D-WT mice^55^. Likewise, subsets of NK cells could be functionally defective, not only in their TRAIL-mediated killing pathway but also in their perf/Gz pathway due to the elevated amount of IL-6 in the tumour milieu^56^.

In contrast with observations in human cancer^57^, we and others have no evidence of NKG2D expression on murine tumour cells themselves in the various models studied^4^,^57^ underlying that the pro-tumorigenic effect of NKG2D in this model is mediated by immune cells. Future studies using conditional NKG2D-deficient mice will help dissect the function of each NKG2D-expressing cell type and determine which ones act as tumour promoters, as tumour suppressors and which cells demonstrate a dual functionality over time, as we predict for CD8^+^ T effector cells.

The present study is highly relevant to human HCC, where NKG2D ligands are expressed as membrane bound and soluble proteins^58^,^59^,^60^,^61^. Our conclusions are in line with recent clinical reports showing that NKG2D is upregulated on hepatic CD8^+^T cells in patients with chronic viral hepatitis^62^ (Prof. M. Maini, personal communication) and that HCV-infected HCC patients display a high density of liver infiltrating CD8^+^T cells which correlated with a higher tumour recurrence and lower survival^63^.

Our findings have direct implications in cancer immunotherapy where NKG2D-based strategies are the focus of intense research^64^,^65^,^66^,^67^,^68^ and a phase I trial for NKG2D CAR T-cell therapy is currently recruiting patients. Enhancing NKG2D-mediated responses in inflammation-driven cancer patients could be deleterious in maintaining a prolonged stimulation that favors tumour growth over rejection. In addition, severe autoimmune reactions may arise from such therapy via targeting healthy tissue expressing NKG2D ligands and/or via cytokine release, as recently evidenced in a preclinical animal model of NKG2D-ligand targeted CAR-therapy^69^. The negative influence of NKG2D in cancer-linked to inflammation unravels a conceptual shift advocating for a careful evaluation of the benefit of NKG2D-based therapy in certain types of cancer.

## Methods

### Mice

*NKG2D-deficient (Klrk1*^*−/−*^) *and NKG2D-sufficient (Klrk1*^*+/+*^) mice on a *Ncr1*^*gfp/+*^ C57BL/6 background^70^ were genotyped using the following primers for *Klrk1*: L1: CAAGTAGTGTGCATTTCATTCAG; P3: ATTGCTCCCTGTCTCATTGTCTT; and WT3: CAGAGCAAGCTTCCTGTTTGTCTCA; and for *NCR1*: NCR REV: AGGAGTTGCCTTCAGTTCCA; NCR FOR: TTGGTCTGGCATGCATAATC; GFP FOR: GCAAAGACCCCAACGAGAAG. Mice were bred and maintained at the Imperial College London's animal facility, in a specific pathogen-free environment. The health status was regularly monitored, blindly, throughout the study. All animal work was carried out in compliance with the British Home Office Animals Scientific Procedures Act 1986 (Project licence number 70/7129).

### HCC induction

Cohorts of male age-matched wild-type (WT) and NKG2D-KO (*Klrk1*^*−/−*^) mice received a single intraperitoneal injection of diethylnitrosamine (DEN) (Sigma) (25 mg kg^−1^ body weight) or PBS at 14–21 days of age to induce HCC. Mice were killed at 15 months of age. Tumour burden was quantified by measurements of the liver/body weight ratio and measurement of tumour diameters that were >5 mm in diameter. Tumour load corresponds to the sum of each tumour diameter over 5 mm. Alanine transaminase (ALT) release into the serum was quantified as an indicator of liver injury (ID Labs, London, Canada). Mice that became terminally ill prior to the endpoint were humanely killed; those presenting a tumour burden at necropsy were included in the survival study.

### Histology and immunohistochemistry

Liver tissues were fixed in 10% neutral buffered formalin, paraffin embedded and cut into 4 μm sections. Sections were H&E stained and assessed for tumour grade by pathologist Professor Robert Goldin (Imperial College London). Necrosis was determined by TUNEL (terminal deoxynucleotidyl transferase-mediated dUTP nick-end labelling) staining (ApopTag Plus Peroxidase *In Situ* Apoptosis Detection Kit, Milipore). Hepatocyte proliferation was determined by staining with polyclonal rabbit anti-MCM4 antibody at a 1/100 dilution (Abcam, ab84153). PD-1 expressing lymphocytes were identified using a rabbit polyclonal anti PD-1 antibody at 2.5 μg ml^−1^ (Biorad ahp1706). RAE-1 expression was analysed using a polyclonal goat anti-mouse RAE-1 antibody at 10 μg ml^−1^ (R&D Systems). HCC markers were stained using anti-Hsp70 at 1 μg ml^−1^ (EPR16892) and rabbit polyclonals raised against Glypican-3 at 5 μg ml^−1^ (ab66596) and Anti-Glutamine Synthetase at 1 μg ml^−1^ (ab73593) from abcam. Slide images were captured using a Nanozoomer slide scanner (Hamamatsu) and analysed using Image J software.

### Tissue dissociation and flow cytometry

Tumour and surrounding regions were delineated macroscopically. Tissues were dissociated through 100 μm cell strainers in PBS with 3% bovine serum albumin (BSA). Hepatocytes were removed by centrifugation on a 35% percoll gradient at 700*g* at 21 °C for 20 min. Leucocytes present in the pellet were resuspended in red blood cell lysis buffer (0.15 M NH_4_Cl, 0.1 mM KHCO_3_, 0.1 mM Na_2_-EDTA in water; pH 7.2) for 1 min, washed and resuspended in PBS 3% BSA. When indicated cells were stimulated with phorbol myristate acetate (PMA) (100 ng ml^−1^) and Ionomycin (1 μg ml^−1^) (Sigma) or plate bound CD3 crosslinking Ab in the presence of Brefeldin A (Sigma) for 4 or 16 h respectively at 37 °C. CD3 crosslinking Ab 2.5 μg ml^−1^ (17A2 Biolegend) was adhered to high binding capacity 96 well plates at overnight at 4 °C. Cell suspensions were incubated with anti-mouse CD16/CD32 (Becton Dickinson, BD, USA) to block Fc receptors and Fixable Viability Dye eFluor 506 (eBioscience, San Diego, CA, USA). Cells were then stained with a cocktail of directly conjugated and biotinylated mAbs (Supplementary Table 3) for 30 min at 4 °C followed by Qdot 605 conjugated streptavidin (Life Technologies, USA) to reveal biotinylated antibodies. Intracellular staining was performed with Cytofix/Cytoperm kit (Becton Dickinson, BD, USA) or with a transcription factor staining buffer set from eBioscience (San Diego, CA, USA). The relevant fluorescence-minus-one labelling conditions including the appropriate isotype mAb were used as controls. All samples were acquired on an LSRFortessa flow cytometer (BD) and analysed with FlowJo version 9.3.1 or above (TreeStar, Ashland, OR, USA).

### MULT1 ELISA

Plates were coated with 2 μg ml^−1^ of MULT1 capture antibody (clone 237104, R&D) overnight at 4 °C. Plates were washed three times with PBST (PBS 0.05% Tween 20) between all incubations. Remaining plate binding capacity was blocked with 1% BSA-PBST. Samples, or recombinant MULT1 (R&D), were diluted in 1% BSA-PBST and incubated overnight at 4 °C. Biotinylated MULT1 detecting antibody (clone 1D6) was incubated at 1 μg ml^−1^ at room temperature for 2 h. HRP-conjugated streptavidin 100 ng ml^−1^ was incubated for 1 h at room temperature (ThermoFisher). After the final wash, 100μl of TMB High Sensitivity HRP Substrate Solution (Biolegend) was added to each well, incubated for 20 min at room temperature. Reaction was quenched by addition of 50 μl of 2M H_2_SO_4_ and absorbance at 450 nm measured using a FluoStar Omega plate reader (BMG Labtech). Sera from male *ApoE*^*−/−*^ mice were fed on a high fat diet for two months, starting at the age of six weeks^19^ and were kindly provided by Prof. Na Xiong. The 1D6 antibody was kindly provided by Prof Stipan Jonjić and biotinylated using EZ-Link Sulfo-NHS-Biotin kit (ThermoFisher).

### RNA isolation and quantitative RT-PCR

Tumour and surrounding liver tissue were collected in RNA-later (Sigma) and stored at −80 °C as per manufacturer's instructions. RNA was extracted using Qiagen's RNeasy kit (Hilden, Germany) and reverse transcribed into cDNA with High capacity cDNA RT kit (Life Technologies). For some genes an amplification step was performed using the TaqMan PreAmp Master Mix Kit (Applied Biosystems). Quantitative real time PCR was carried out using the TaqMan system (Applied Biosystems), all values were normalized to GAPDH expression. The list and identification number of each Taqman probes is shown in Supplementary table 4.

### Statistical analysis

Statistical analyses were performed using GraphPad Prism software version 5.03 (GraphPad Software Inc.). Log-rank (Mantel-Cox) test was applied to survival analysis. Two-tailed unpaired Student's *t*-test and Mann–Whitney tests were applied (as indicated in the figure legend) when appropriate according to the Shapiro–Wilk normality test. Welch's correction was applied to *t*-tests when appropriate. Correlation was established based on linear regression analysis. Differences at *P*≤0.05 were considered significant. *=*P*≤0.05, **=*P*≤0.01, ***=*P*≤0.001, ****=*P*≤0.0001.

### Data availability

The authors declare that all the data supporting the findings of this study are available within the article and its Supplementary Information files and from the corresponding author on reasonable request.

## Additional information

**How to cite this article:** Sheppard, S. *et al*. The immunoreceptor NKG2D promotes tumour growth in a model of hepatocellular carcinoma. *Nat. Commun.* **8,** 13930 doi: 10.1038/ncomms13930 (2017).

**Publisher's note:** Springer Nature remains neutral with regard to jurisdictional claims in published maps and institutional affiliations.

## Supplementary Material

Supplementary Information Supplementary Figures and Supplementary Tables.
