# Supplementary material for: The immunoreceptor NKG2D promotes tumour growth in a model of hepatocellular carcinoma
Source: Nat Commun. 2017 Jan 27;8:13930. doi: 10.1038/ncomms13930 (PMC5290164; doi:10.1038/ncomms13930)
Supplement: Supplementary Information — Supplementary Figures and Supplementary Tables. [file ncomms13930-s1.pdf]

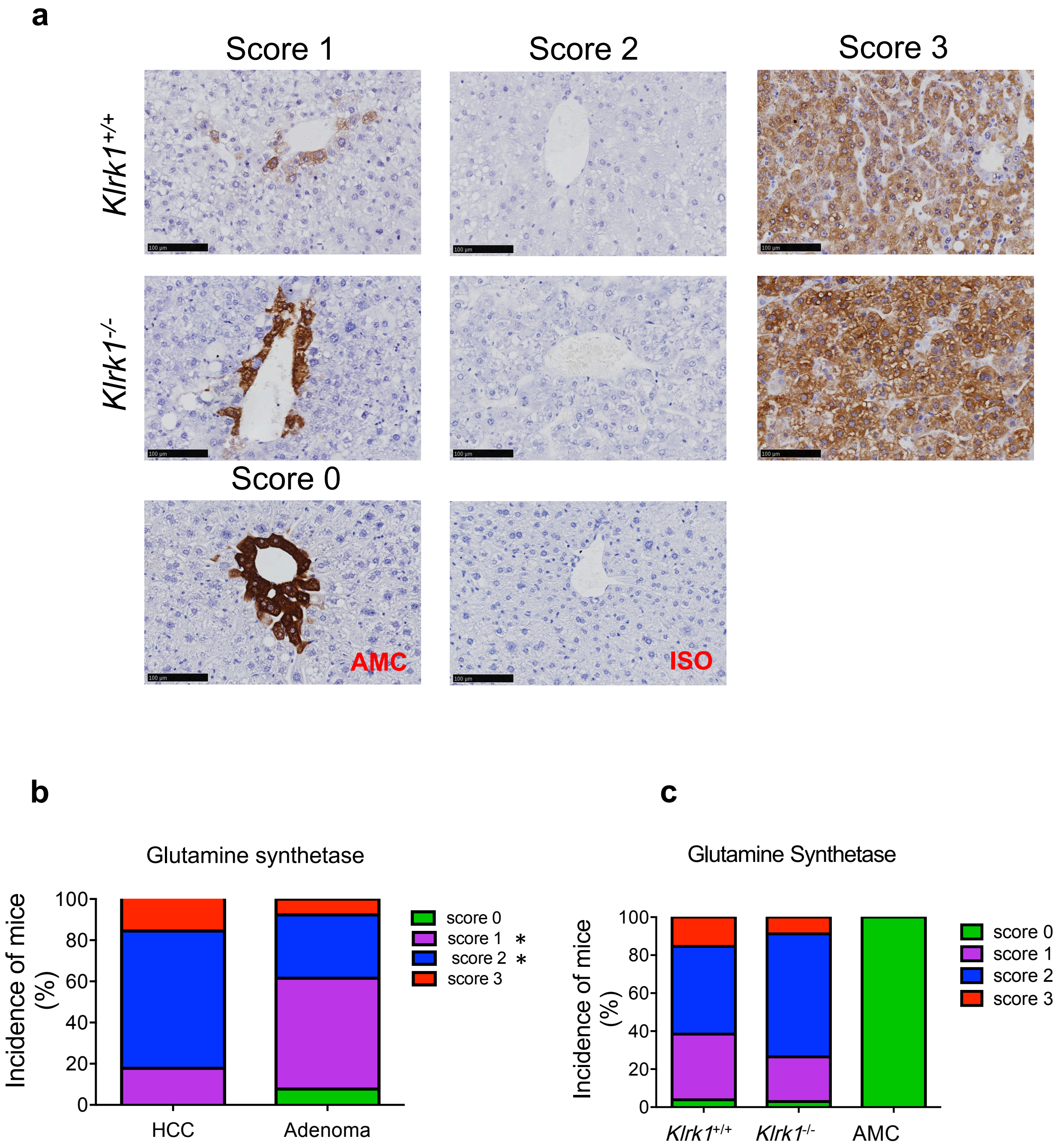

a
Score 1
Score 2
Score 3
Klrk1+/+
Klrk1-/-
Score 0
AMC
ISO
b
Glutamine synthetase
Incidence of mice (%)
100
80
60
40
20
0
HCC
Adenoma
score 0
score 1 *
score 2 *
score 3
c
Glutamine Synthetase
Incidence of mice (%)
100
80
60
40
20
0
Klrk1+/+
Klrk1-/-
AMC
score 0
score 1
score 2
score 3

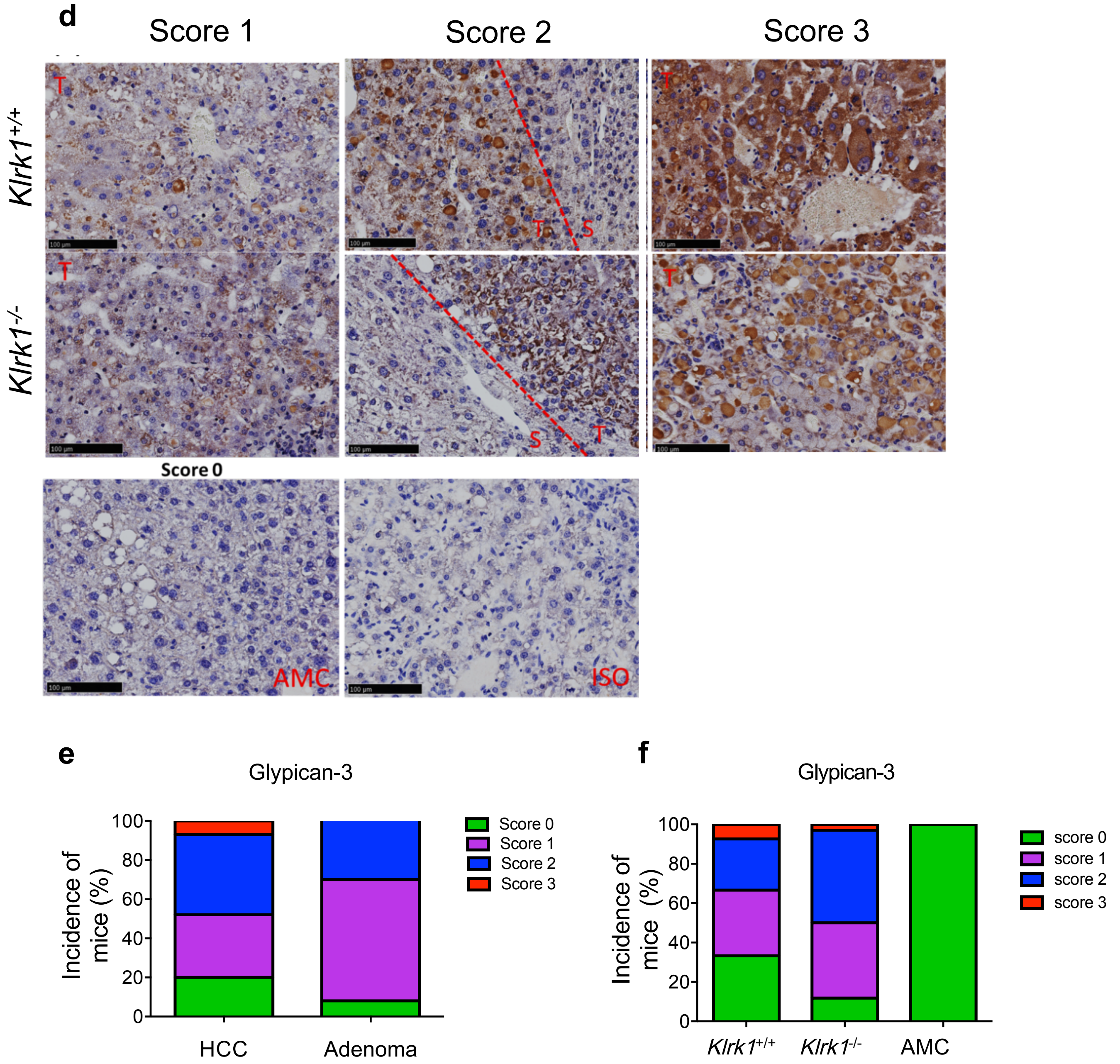
d
Score 1
Score 2
Score 3
Klrk1+/+
Klrk1-/-
T
S
Score 0
AMC
ISO
e
Glypican-3
Incidence of mice (%)
100
80
60
40
20
0
HCC
Adenoma
Score 0
Score 1
Score 2
Score 3
f
Glypican-3
Incidence of mice (%)
Klrk1+/+
Klrk1-/-
AMC
score 0
score 1
score 2
score 3

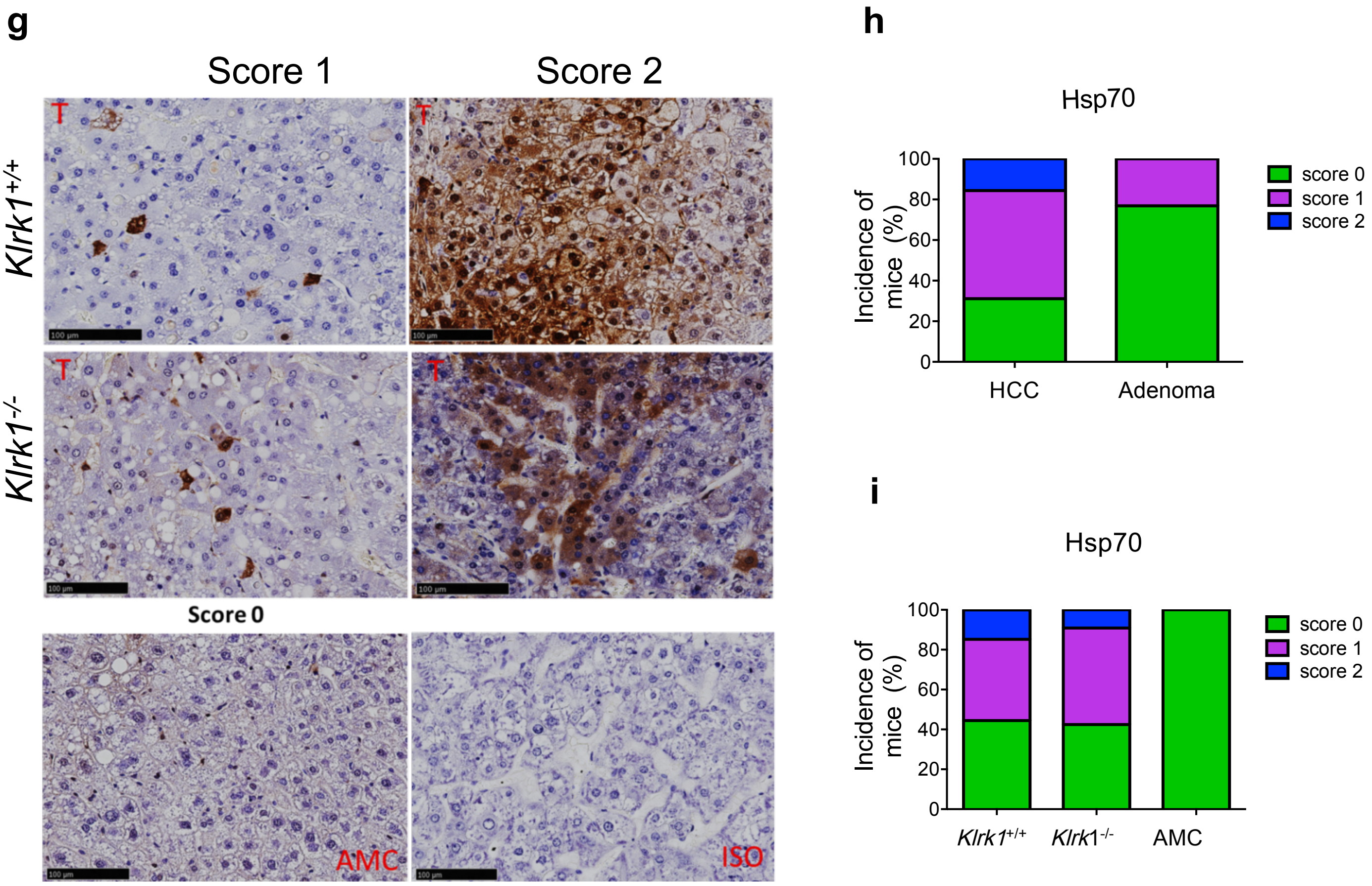

Supplementary Figure 1. Expression analysis of HCC markers on tumors from DEN-treated *Klrk1*$^{+/+}$ (n=30) and *Klrk1*$^{-/-}$ (n=34) mice.

(a, d, g) Representative immunohistochemical staining of Glutamine Synthetase (a), Glypican-3 (d) and Hsp70 (g). Images correspond to representative tissue section of a normal liver (age-matched control AMC) and tissue sections of the tumor (T) and surrounding tissue (S) from DEN-treated Klrk1$^{+/+}$ and Klrk1$^{-/-}$ mice. Scores were individually established based on the extent of staining (% area) for each staining (Supplementary table 2). The red line demarcates boundary between tumour and surrounding area. Scale bar represents 100 µm. (b, e, h) Comparison of Glutamine Synthetase (b), Glypican-3 (e) and Hsp70 (h) expression between carcinomas (HCC) and non-malignant adenomas (pre-HCC). (c, f, i) Comparision of the Glutamine Synthetase (c), Glypican-3 (f), Hsp70 (i) expression scores in *Klrk1*$^{+/+}$, *Klrk1*$^{-/-}$ and AMC mice. Statistical analysis was performed used Fisher's exact test. * indicate P-values ≤0.05.

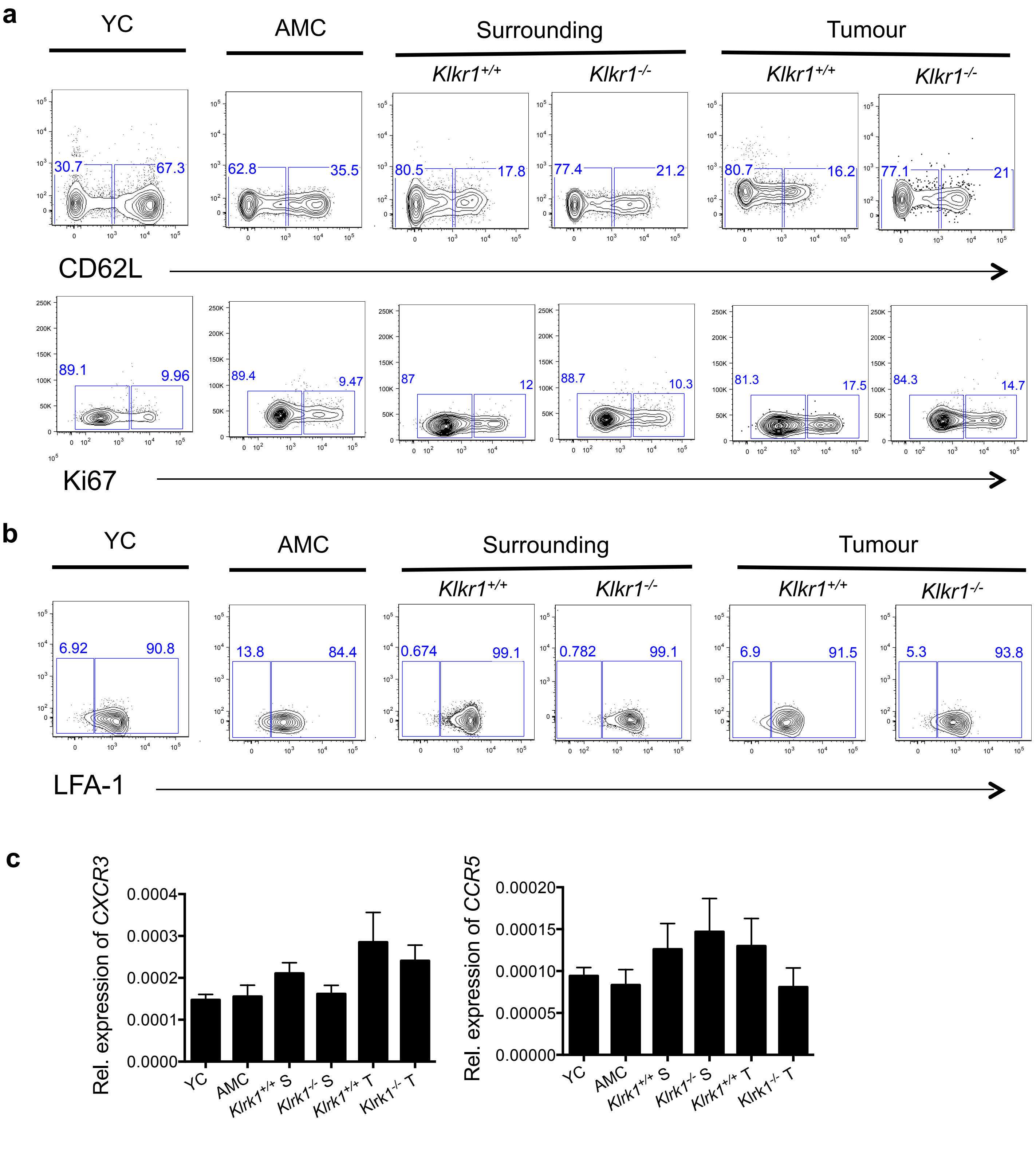

Supplementary Figure 2. A large majority of CD8+T cells in DEN-treated mice are memory cells.

(a) Representative flow cytometry plots of CD62L and Ki67 expression on $CD8^{+}$T cells ($CD45^{+}CD3^{+}NK1.1^{-}CD8^{+}$) and (b) LFA-1 on $CD62L^{-}CD8^{+}$T, that surround (S) and infiltrate the tumours (T), of *Klrk1*$^{+/+}$ and *Klrk1*$^{-/-}$ DEN-treated mice and untreated age-matched control mice (AMC) (representative of n≥3). (c) Quantification of CXCR3 and CCR5 mRNA transcripts within tumors (T) and surrounding (S) liver tissues of *Klrk1*$^{+/+}$ (n≥17) and *Klrk1*$^{-/-}$ (n≥15) DEN-treated mice and from AMC (n≥9) and YC (n≥5). Bar graphs represent the mean ± SEM. Statistical analysis was performed by unpaired Student's t test.

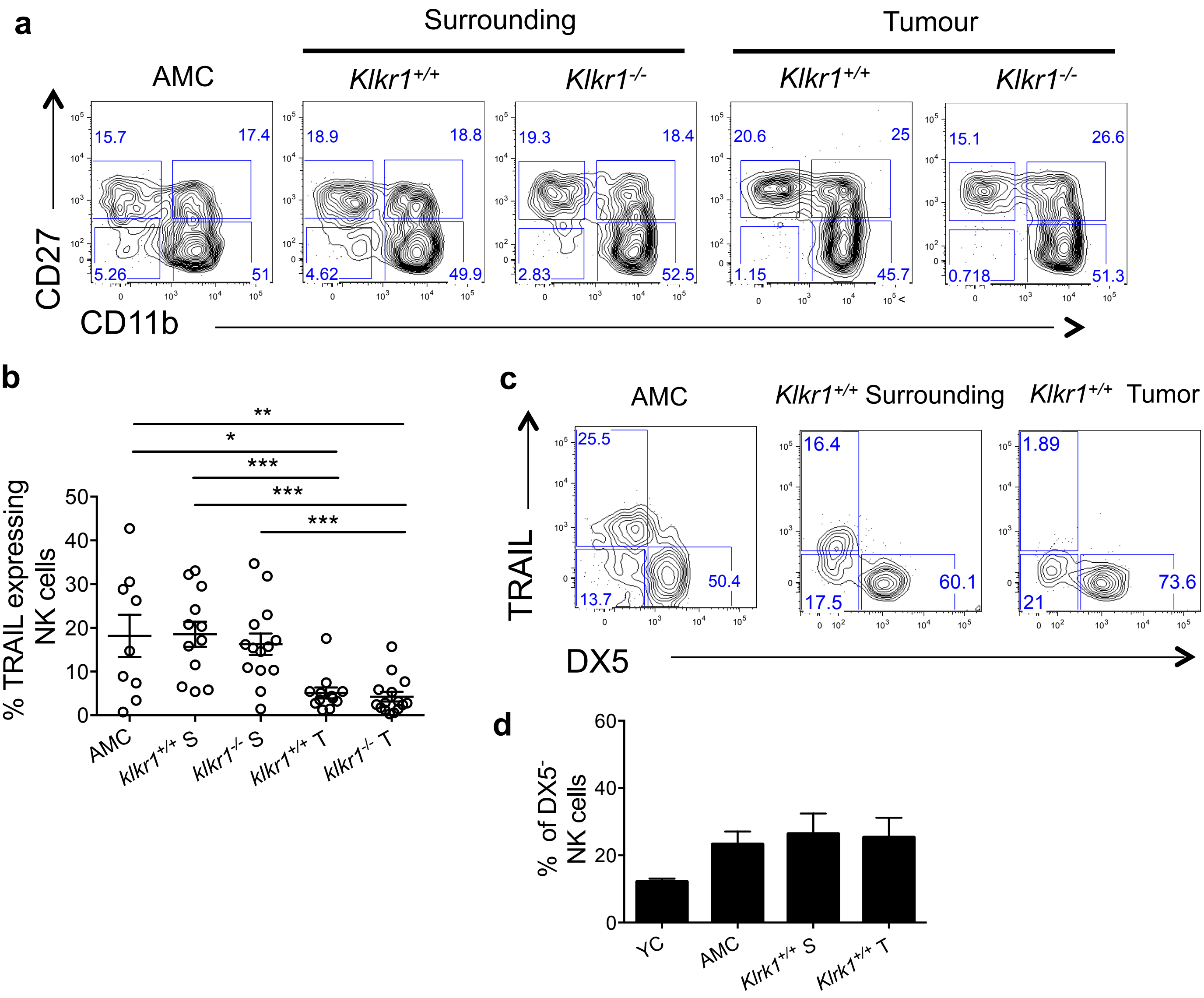

Supplementary Figure 3. Tumor infiltrating NK cells exhibit a drastic reduction in TRAIL expression

(a) Representative flow cytometry plots of CD27 vs CD11b expression on NK cells (CD3- NK1.1+ NKp46+) that surround and infiltrate the tumor of *Klkrk1*+/+ and *Klrk1*-/- DEN-treated mice and untreated age-matched control mice (AMC) (n≥5). CD11b and CD27 dissect NK cells into four populations listed here in order of increasing maturity from left to right CD11b^{lo}CD27^{hi} < CD11b^{lo}CD27^{hi} < CD11b^{hi}CD27^{hi} < CD11b^{hi}CD27^{lo}. (b) Percentages of TRAIL expressing NK cells (CD3$^-$NK1.1$^+$NKp46$^+$ DX5$^-$) from the tumour (T) and surrounding tissue (S) of 15-month-old *Klrk1*$^{+/+}$ and *Klrk1*$^{-/-}$ DEN-treated and untreated age-match control mice (AMC) (n≥6). Dots represent individual mouse; the mean ± SEM are depicted as horizontal and whiskers lines respectively. Statistical analysis was performed by unpaired Student's t test, ; * = p≤0.05, ** = p≤0.01, ***= p≤0.001. (c) Representative flow cytometry plots of DX5 vs TRAIL expression on NK cells (n≥3) (d) percentages of DX5 -negative liver NK cells (n≥3 for each group) surrounding (S) and infiltrating the tumour (T) of *Klrk1*$^{+/+}$ DEN-treated mice and untreated age-matched control mice (AMC) and young controls (YC). Bar graph represents the mean ± SEM. Statistical analysis was performed by unpaired Student's t test; * = p≤0.05, ** = p≤0.01, ***= p≤0.001.

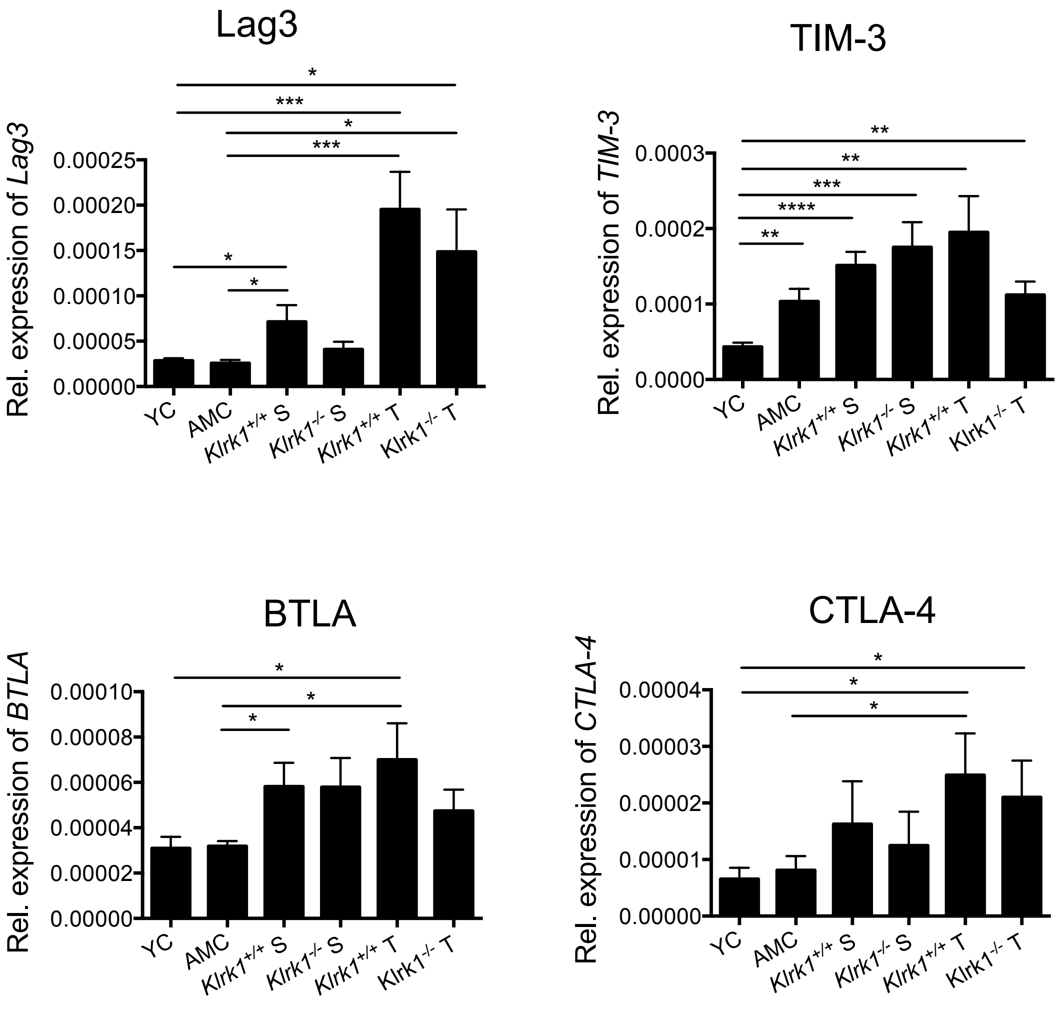

Supplementary Figure 4. Gene expression analysis of Inhibitory receptors.

Relative expression of Lag3, TIM-3, BTLA and CTLA-4 transcripts within tumour (T) and surrounding (S) liver tissue of *Klrk1*$^{+/+}$ (n≥20) and *Klrk1*$^{-/-}$ (n≥18) DEN-treated mice and from age-match control (AMC) (n≥18). Bar graphs represent the mean ± SEM. Statistical analysis was performed by unpaired Student's t test; * = p≤0.05, ** = p≤0.01, *** = p≤0.001.

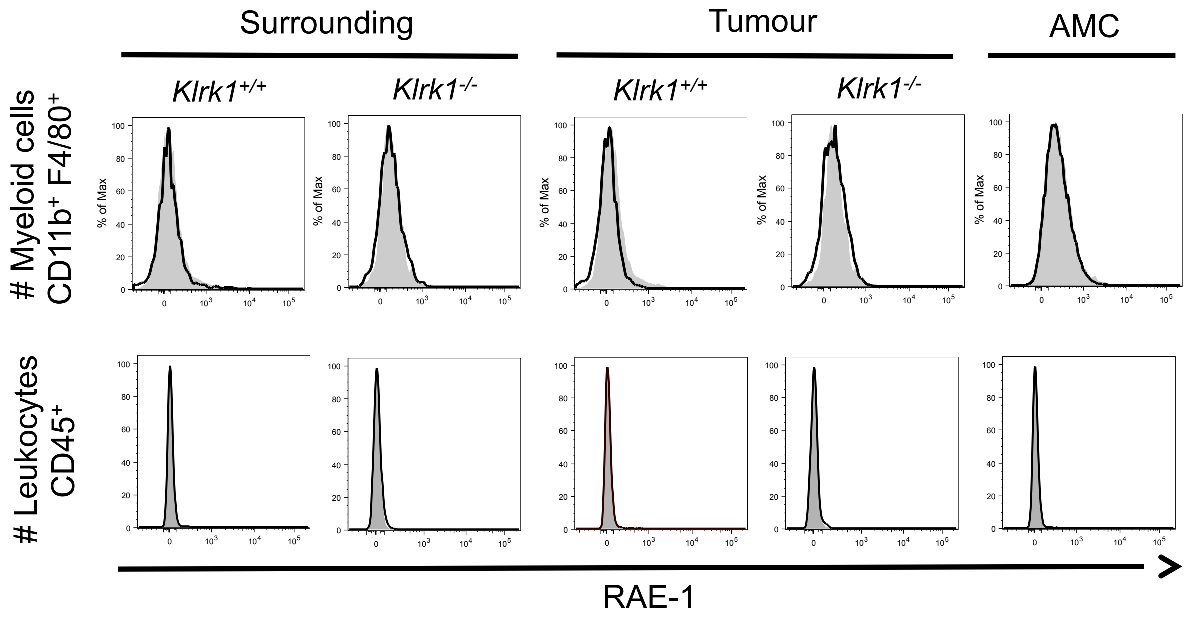

Supplementary Figure 5. NKG2D ligand RAE-1 is not detected on CD11b$^+$F4/80$^+$ myeloid cells or leukocytes.

Representative flow cytometry histograms of pan-RAE-1 staining on CD11b$^+$F4/80$^+$ cells (top black lines) and on CD45$^+$ cells (bottom black lines) that surround and infiltrate the tumour of *Klrk1$^{+/+}$* and *Klkr1$^{-/-}$* DEN-treated mice and untreated age-matched control mice (AMC), fluorescence minus ones control in grey filed histogram (representative of n≥6).

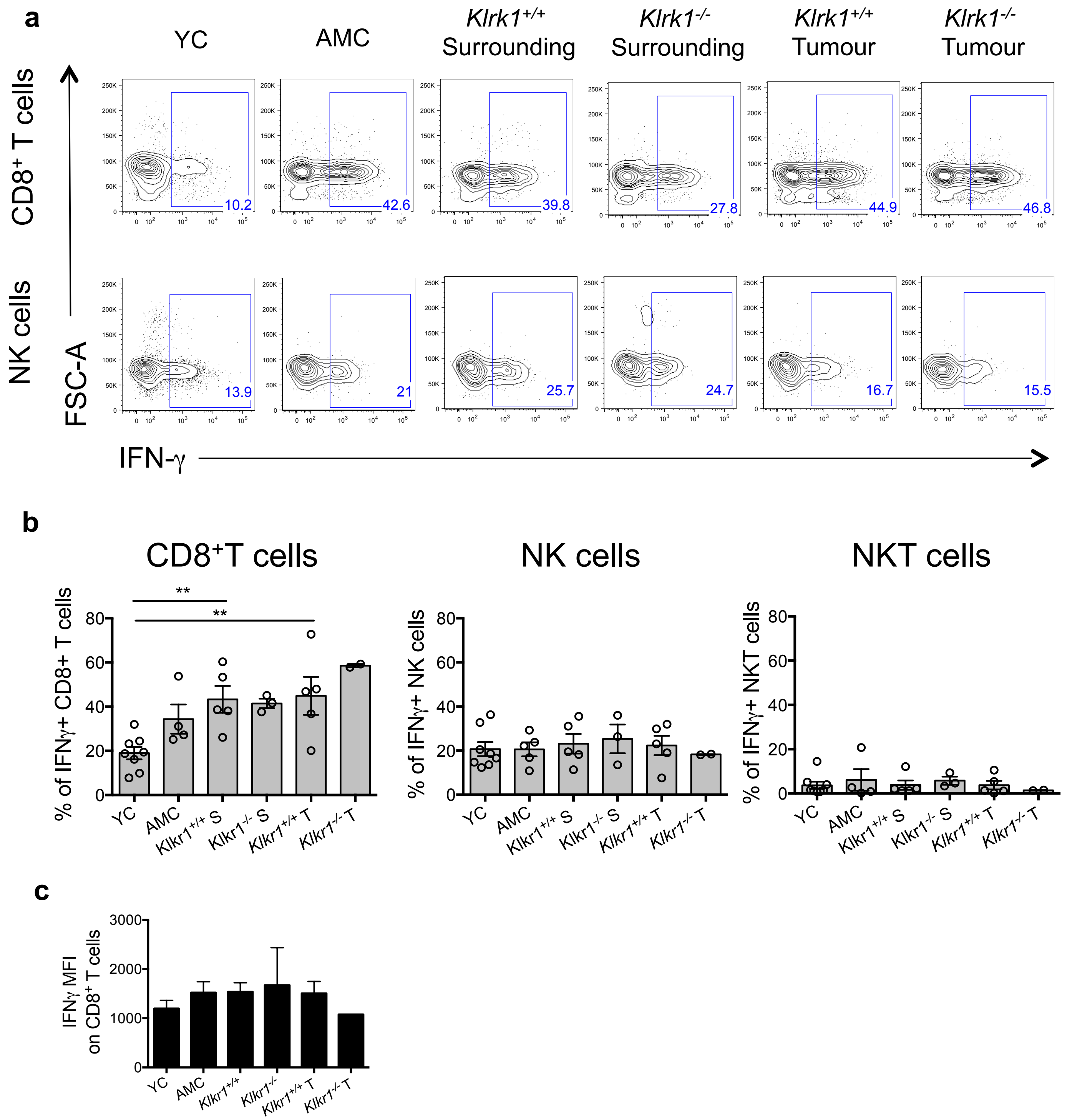

Supplementary Figure 6. The activation potential of CD8$^+$T cells but not NK cells is increased in the livers of 15 month old mice.

(a) Representative flow plots of anti-IFNγ stained CD8$^+$T cells and NK cells post 4-hour re-stimulation with phorbol myristate acetate (PMA) and Ionomycin. (b) Percentages of IFNγ positive CD8+T cells, NK cells and NKT cells from the tumour (T) and surrounding tissue (S) of 15-month-old Klrk1$^{+/+}$ and Klrk1$^{-/-}$ DEN-treated, untreated age-match control mice (AMC) or young controls (YC) post 4-hour re-stimulation with PMA and Ionomycin. Dots represent individual mouse and mean ± SEM are depicted on each bar (c) Bar graph representing the median fluorescence intensity (mean ± SEM) of anti-IFNγ stained CD8$^+$T cells post 4-hour re-stimulation with PMA and Ionomycin.

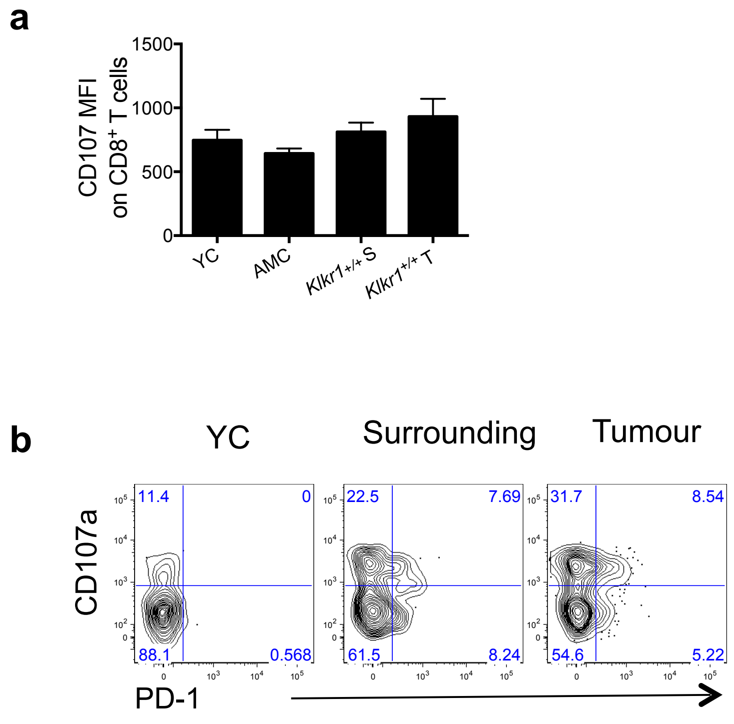

Supplementary Figure 7. CD107a expression on CD8^{+}T cells from DEN-treated NKG2D-WT mice.

(a) Median fluorescence intensity of CD107a on freshly isolated CD8+T cells from *Klrk1*$^{+/+}$ mice. Bars represent mean ± SEM. Statistical analysis was performed by unpaired Student's t test; * = p≤0.05, ** = p≤0.01, *** = p≤0.001. (b) Representative flow plots of PD-1 and CD107a expression on CD8+T cells from *Klrk1*$^{+/+}$ mice assessed post in vitro stimulation with plate-bound anti-CD3 antibody.

Supplementary Table 1: Histopathological features of tumors harvested from 15 month old *Klrk1*$^{+/+}$ and *Klrk1*$^{-/-}$ DEN-treated mice

Type of Lesion *Klrk1+/+* cohort (nb of mice) %	IHC score	GS	Glypican-3	Hsp70	HCC Grade	Avg Tumor Size (±SEM)	Avg Liver/Body Weight Ratio (±SEM)
**HCC** (22/30) **73.3%**	0	0.0%	33.3%	38.1%	**GRADE 1** (1/22) **4.5%**	18	0.13
	1	23.8%	33.3%	42.9%	**GRADE 2** (10/22) **45.5%**	17.9 (±1.345)	0.141 (±0.011)
	2	57.2%	23.8%	19.0%			
	3	19.0%	9.6%	N/A	**GRADE 3** (11/22) **50.0%**	20.4 (±1.770)	0.153 (±0.014)
**Adenoma** (6/30) **20.0%**	0	0.0%	0.0%	66.7%	N/A	4.8 (±0.401)	0.055 (±0.002)
	1	60.0%	50.0%	33.3%			
	2	40.0%	50.0%	0.0%			
	3	0.0%	0.0%	N/A			
**Nodule** (2/30) **6.7%**	N/A	N/A	N/A	N/A	N/A	2.5 (±0.5)	0.050 (±0.000)

N/A = not applicable; GS = Glutamine Synthetase

Type of Lesion *Klrk1-/-* cohort (nb of mice) %	IHC score	GS	Glypican-3	Hsp70	HCC Grade	Avg Tumor Size (±SEM)	Avg Liver/Body Weight Ratio (±SEM)
**HCC (24/34) 70.6%**	0	0.0%	11.1%	25.9%	**GRADE 1** (1/24) **4.2%**	8	0.083
	1	14.8%	29.6%	63.0%	**GRADE 2** (15/24) **62.5%**	17.1 (±1.380)	0.120 (±0.010)
	2	74.1%	55.6%	11.1%			
	3	11.1%	3.7%	N/A	**GRADE 3** (8/24) **33.3%**	16.1 (±1.476)	0.111 (±0.011)
**Adenoma** (8/34) **23.5%**	0	14.3%	14.3%	85.7%	N/A	4 (±0.655)	0.056(±0.003)
	1	57.1%	71.4%	14.3%			
	2	28.6%	14.3%	0.0%			
	3	0.0%	0.0%	N/A			
**Nodule** (2/34) **5.9%**	N/A	N/A	N/A	N/A	N/A	4.5 (±1.5)	0.051 (±0.001)

N/A = not applicable; GS = Glutamine Synthetase

Supplementary Table 2: Scoring system based on IHC staining for Glutamine Synthetase, Heat shock protein 70 and Glypican-3
(as previously reported (1))

Marker	Score	Description
**Glutamine Synthetase (GS)**	0	Normal- All perivenous hepatocytes are positive for GS
	1	A few perivenous hepatocytes remain positive
	2	No hepatocytes stain positive
	3	Large region of positive hepatocytes
**Heat shock protein 70 (Hsp70)**	0	Highly infrequent hepatocyte immunoreactivity
	1	Focal immunoreactivity (i.e. dispersed solitary positive hepatocytes spread throughout the tumor)
	2	Diffuse immunoreactivity, i.e. patchy staining of areas of hepatocytes
**Glypican-3 (GPC3)**	0	No hepatocytes stain positive
	1	Infrequent hepatocyte immunoreactivity
	2	A greater proportion of hepatocytes stain positive
	3	Large region of positive hepatocytes

Supplementary Table 3. List of antibodies used for flow cytometry

Antigen	Clone	Concentration (μg/ml)	Fluorophore	Supplier
CD3	17A2/ 145-2C11	2	V450/BV421/FITC/ APC-Cy7/BV605	BD/ BioLegend
CD4	RM4-5	0.125	APC-eFluor® 780	eBioscience
CD8a	53-6.7	0.5	AF 647/ PerCP-Cy5.5	eBioscience
CD11b (Mac1)	M1/70	1.33	PerCP-Cy5.5/PE-CF®594	eBioscience/ BD
CD11c	N418	1	PE	eBioscience
CD27	LG.7F9	0.5	PE-Cy7	eBioscience
CD45	30-F11	0.5	Biotin/APC/APC-Cy7/FITC/PE-Cy7/BV605	eBioscience/ BioLegend
CD62L	MEL-14	0.125	PE-CF®594	BD
CD69	H1.2F3	0.63	Biotin	eBioscience
CD107a (Lamp)	1D4B	4	PE-Cy7	BD
Dx5	Dx5	8	PE/APC	eBioscience
F4/80	BM8	4	PE-Cy7	eBioscience
Gr-1	RB6-8C5	2	APC-Cy7	BioLegend
IFNγ	XMG1.2	2	PE	eBioscience
Ki67	SolA15	2	eFluor® 450	eBioscience
LFA-1	H155-78	0.5	PerCP-Cy5.5	BioLegend
Ly6C	HK1.4	0.63	Biotin	BioLegend
Ly6G	1AB	2	APC-Cy7	BioLegend
NK 1.1	PK135	1	PE-Cy7/AF 488	BioLegend
NKG2D	A10/CX5	2	Biotin	eBioscience
NKp46	29A1.4	4	APC/FITC	BioLegend
PD-1	J43	2	PE-CF®594	BD
Rae1 (pan)	186107	n/a (dilution1/100)	APC	R&D
TRAIL	N2B2	5	Biotin	eBioscience

Supplementary Table 4. List of primers used for qRT-PCR with the Taqman system

Target gene	Taqman primers ID
IL-6	Mm00446190_m1
GAPDH	Mm99999915_g1
CCL5	Mm01302427_m1
CCL3	Mm00441259_g1
IFN-γ	Mm01168134_m1
TNF-α	Mm00443258_m1
CXCL10	Mm00445235_m1
IL-10	Mm00439614_m1
IL-18	Mm00434226_m1
CXCL9	Mm00434946_m1
IL-15	Mm00434210_m1
IL-33	Mm00505403_m1
IL-25	Mm00840829_m1
CCR2	Mm99999051_gH
TGFβ	Mm01178820_m1
MMP14	Mm00485054_m1
ADAM10	Mm00545742_m1
MMP9	Mm00442991_m1
GZMB	Mm00442837_m1
PD1	Mm01285676_m1
KLRK1	Mm00473603_m1
CD274	Mm00452054_m1
CDKN1a	Mm04205640_g1
PRF1	Mm00812512_m1

Supplementary Reference.

1. Di Tommaso, L. *et al*. Diagnostic value of HSP70, glypican 3, and glutamine synthetase in hepatocellular nodules in cirrhosis. *Hepatology* **45**, 725-734, doi:10.1002/hep.21531 (2007)
